# Mitochondrial Disease and the Kidney With a Special Focus on CoQ_10_ Deficiency

**DOI:** 10.1016/j.ekir.2020.09.044

**Published:** 2020-10-10

**Authors:** Anne M. Schijvens, Nicole C. van de Kar, Charlotte M. Bootsma-Robroeks, Elisabeth A. Cornelissen, Lambertus P. van den Heuvel, Michiel F. Schreuder

**Affiliations:** 1 Department of Pediatric Nephrology, Radboud University Medical Center, Radboud Institute for Molecular Life Sciences, Amalia Children’s Hospital, Nijmegen, the Netherlands; 2 Department of Development and Regeneration,University Hospital Leuven, Leuven, Belgium

**Keywords:** coenzyme Q10, mitochondrial kidney disease, nephrotic syndrome

## Abstract

Mitochondrial cytopathies include a heterogeneous group of diseases that are characterized by impaired oxidative phosphorylation, leading to multi-organ involvement and progressive clinical deterioration. Most mitochondrial cytopathies that cause kidney symptoms are characterized by tubular defects, but glomerular, tubulointerstitial, and cystic diseases have also been described. Mitochondrial cytopathies can result from mitochondrial or nuclear DNA mutations. Early recognition of defects in the coenzyme Q_10_ (CoQ_10_) biosynthesis is important, as patients with primary CoQ_10_ deficiency may be responsive to treatment with oral CoQ_10_ supplementation, in contrast to most mitochondrial diseases. A literature search was conducted to investigate kidney involvement in genetic mitochondrial cytopathies and to identify mitochondrial and nuclear DNA mutations involved in mitochondrial kidney disease. Furthermore, we identified all reported cases to date with a CoQ_10_ deficiency with glomerular involvement, including 3 patients with variable renal phenotypes in our clinic. To date, 144 patients from 95 families with a primary CoQ_10_ deficiency and glomerular involvement have been described based on mutations in *PDSS1*, *PDSS2*, *COQ2*, *COQ6*, and *COQ8B/ADCK4*. This review provides an overview of kidney involvement in genetic mitochondrial cytopathies with a special focus on CoQ_10_ deficiency.

Mitochondrial cytopathies include a heterogeneous group of diseases with impaired oxidative phosphorylation.^1^ Mitochondrial disorders are characterized by a progressive clinical deterioration of the disease and the presence of some degree of encephalopathy in most patients. However, other organs with high metabolic rates can also be severely affected.^2^ The kidney is an energy-demanding organ in the human body and all nephron segments have different mitochondrial densities and distributions due to different energy demands along the nephron.^3^, ^4^, ^5^ Kidney symptoms caused by mitochondrial dysfunction are often characterized by tubular defects, however, glomerular, tubulointerstitial, and cystic diseases have also been described.^6^ Moreover, kidney manifestations are rarely isolated and often part of a multisystemic disorder with symptoms of encephalopathy, cardiomyopathy, sensorineural hearing loss, muscle weakness, retinopathy, and/or diabetes mellitus.^2^ Mitochondrial cytopathies are caused by inherited or sporadic mitochondrial deoxyribonucleic acid (mtDNA) or nuclear DNA (nDNA) mutations in genes that affect mitochondrial functions. Disease-causing mutations that result in disorders of mitochondrial energy metabolism have been reported at present in >250 genes.^7^ Although each individual defect is rare, the overall prevalence of mitochondrial disorders in the general population is approximately 1 in 4300.^8^^,^^9^ For mitochondrial diseases with kidney involvement, 2 important genetic causes should be highlighted: defects in the mtDNA3243A>G and in the CoQ_10_ biosynthesis. The m.3243A>G mutation in the transfer ribosomal ribonucleic acid (tRNA)^Leu^ gene is one of the most common mtDNA point mutations. The phenotypic expression of m.3243A>G mutation can be highly variable and cause a wide range of clinical manifestations. Although kidney involvement is not very common, several patients with the m.3243A>G mutation have developed proteinuria and kidney failure.^10^ In patients with primary CoQ_10_ deficiency, proteinuria and/or kidney dysfunction might be the only manifestation at presentation. CoQ_10_ deficiency is highly relevant for clinicians because, in contrast to most mitochondrial disorders, patients with primary CoQ_10_ deficiency may respond to treatment with CoQ_10_ supplementation.^11^ Early diagnosis is crucial, as oral supplementation of CoQ_10_ can limit disease progression, prevent neurological deterioration, and improve clinical symptoms.^12^ As established neurologic and/or kidney damage is irreversible, this underlines the importance of early diagnosis and treatment. In this review, an overview of kidney involvement in genetic mitochondrial cytopathies is provided with a special focus on CoQ_10_ deficiency.

## Mitochondrial (DYS)Function

**Figure 1:**
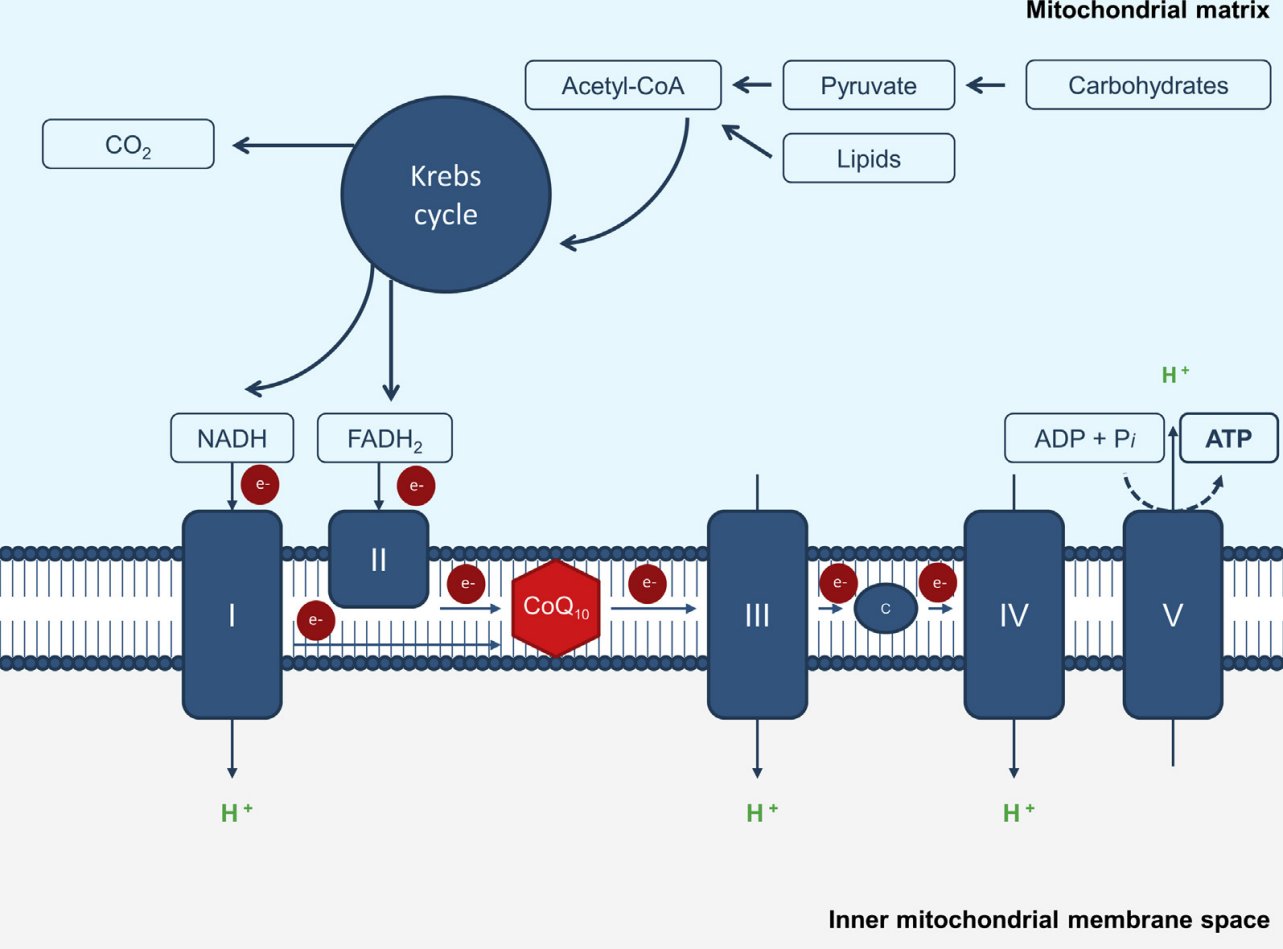
Mitochondrial energy-generating system. Acetyl-CoA, the terminal product of carbohydrate and lipid metabolism, enters the Krebs cycle to generate CO_2_, NADH, and FADH_2_. Electrons derived from cellular dehydrogenases in the Krebs cycle are passed along 4 protein complexes and 2 small carriers. The flow of electrons from NADH or FADH_2_ through the protein complexes leads to the pumping of protons from the mitochondrial matrix to the intermembrane space. The electrons are shuttled from complex I and II to complex III by the electron carrier CoQ_10_ and then transferred to complex IV by cytochrome c. These processes create an electrochemical gradient that is used by ATP synthase (complex V) to synthesize ATP from inorganic phosphate and ADP. Acetyl-CoA, acetyl coenzyme A; ADP, adenosine diphosphate; ATP, adenosine triphosphate; C, cytochrome c; e, electron; FADH_2_, reduced form of flavin adenine dinucleotide; NADH, nicotinamide adenine dinucleotide hydrogen; CoQ_10_, coenzyme Q_10_; I, complex I; II, complex II; III, complex III; IV, complex IV; V, complex V.

The mitochondrial genome is composed of a single, double-stranded, circular loop that lacks introns and contains 37 genes, encoding 2 ribosomal RNA, 22 transfer RNAs, and 13 structural protein subunits of the respiratory chain complexes I, III, and IV, and protein complex V.^13^, ^14^, ^15^ Complex II contains only nDNA-encoded subunits, whereas the other respiratory chain complexes and complex V comprise both mtDNA-encoded and nDNA-encoded subunits.^8^ The mitochondrial respiratory chain is composed of 4 protein complexes and 2 electron carriers: CoQ_10_ and cytochrome c (Figure 1). CoQ_10_, also known as ubiquinone, is present in all cell membranes as a lipid molecule with a variety of biological functions.^16^ Besides its function as electron carrier, CoQ_10_ is involved in the β-oxidation of fatty acids, pyrimidine synthesis, detoxification of hydrogen sulfide, and protection from reactive oxygen species (ROS).^2^^,^^17^, ^18^, ^19^ Mitochondrial cytopathies refer to inherited or sporadic mtDNA or nDNA mutations in genes that affect mitochondrial functions. Different from nDNA, various amounts of mtDNA copies are present in a cell depending on its energy demand. mtDNA is highly susceptible to damage and mutations, with a 10- to 1000-fold greater mutation rate than nDNA.^20^ The threshold level for mitochondria to become dysfunctional can vary between tissues due to differences in energy dependence. The threshold for disease is lower in tissues that are highly dependent on oxidative metabolism, such as kidney tubules.^2^^,^^21^ Defects in oxidative phosphorylation cause 1 major problems: a reduction in ATP production, and an increase in ROS production. In approximately one-third of patients, the first symptoms of mitochondrial defects develop within the first weeks of life, and more than 80% of patients are symptomatic by the age of 2 years.^22^

## Kidney Manifestations of Genetic Mitochondrial Cytopathies

Kidney symptoms caused by mitochondrial dysfunction are often characterized by tubular or glomerular defects. Tubulointerstitial and cystic kidney diseases have also been described; however, the molecular mechanisms remain to be elucidated.^20^

### Tubular Defects

**Figure 2:**
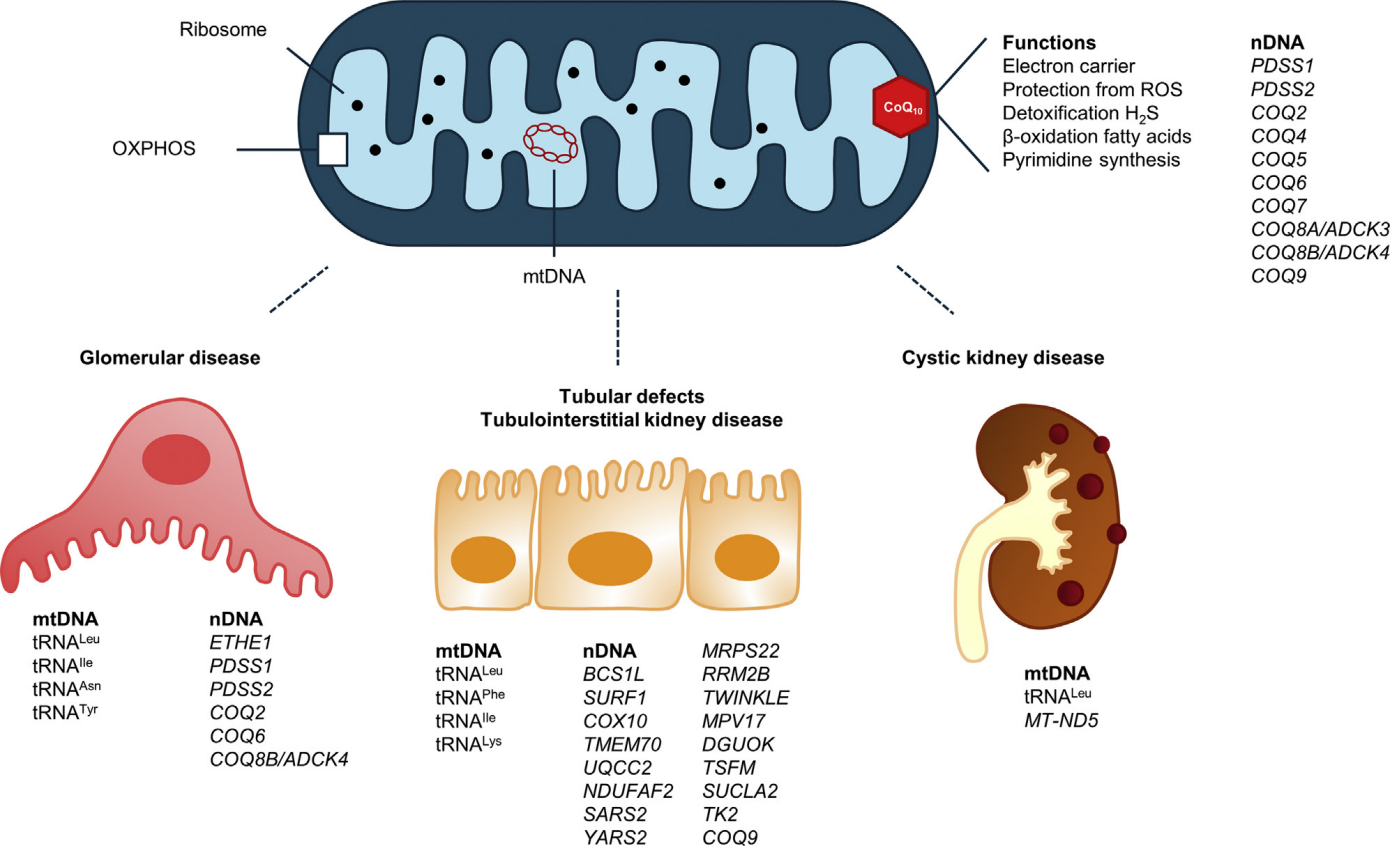
Mitochondrial disease and the kidney. Kidney manifestations reported in the setting of mitochondrial cytopathies include tubular dysfunction, interstitial nephritis, glomerular pathology, and, in rare cases, cystic disease. Various mitochondrial DNA and nuclear DNA mutations are involved, as depicted in Tables 1 to 3. H_2_S, hydrogen sulfide; mt, mitochondrial; n, nuclear; OXPHOS, oxidative phosphorylation; ROS, reactive oxygen species; tRNA, transfer ribonucleic acid.

Tubular disorders are frequently reported in patients with mitochondrial cytopathies. Mutations in both mitochondrial and nuclear genes have been described to cause tubular defects (Tables 1 and 2, Figure 2).^10^^,^^23^, ^24^, ^25^, ^26^, ^27^, ^28^, ^29^, ^30^, ^31^, ^32^, ^33^, ^34^, ^35^, ^36^, ^37^, ^38^, ^39^, ^40^, ^41^, ^42^, ^43^, ^44^, ^45^, ^46^, ^47^, ^48^, ^49^, ^50^, ^51^, ^52^, ^53^, ^54^, ^55^, ^56^, ^57^, ^58^, ^59^, ^60^, ^61^, ^62^, ^63^, ^64^, ^65^, ^66^, ^67^, ^68^, ^69^, ^70^, ^71^ Excellent overviews of mitochondrial and nuclear gene mutations causing tubular defects are provided in various reviews.^8^^,^^20^^,^^72^, ^73^, ^74^ The most commonly reported problem is a proximal tubular defect, as proximal tubular cells are relatively vulnerable to oxidative stress. Most patients present with partial defects, including renal tubular acidosis (RTA), aminoaciduria, glycosuria, hypermagnesuria, or a combination of the above.^8^^,^^73^ Renal Fanconi syndrome has been reported in children with specific mitochondrial syndromes, including Kearns−Sayre syndrome, Pearson syndrome, Leigh syndrome, and CoQ_10_ deficiency.^70^^,^^75^, ^76^, ^77^, ^78^, ^79^ Kearns−Sayre syndrome typically includes chronic progressive external ophthalmoplegia, ptosis, and pigmentary retinopathy in individuals less than 20 years of age.^80^ Pearson syndrome occurs in infancy, and characteristically includes sideroblastic anemia and exocrine pancreatic dysfunction.^81^ Hypomagnesemia has been described in different mitochondrial syndromes, for instance in patients with Kearns−Sayre syndrome.^82^^,^^83^ Moreover, Wilson *et al.* describe a cluster of metabolic defects, including hypomagnesemia, caused by a mitochondrial tRNA^Ile^ mutation.^44^

**Table 1:** Genotype phenotype correlation of mitochondrial DNA mutations involved in mitochondrial cytopathies with kidney involvement

Gene	mtDNA point mutation	Predominant kidney phenotype	Extrarenal symptoms	Ref
tRNA^Leu^	m.3243A>G	FSGS, tubulointerstitial nephritis, cystic kidney disease	Wide range of clinical manifestations including, MELAS syndrome, deafness, diabetes mellitus, neuromuscular involvement, hypertrophic cardiomyopathy, macular dystrophy	10, 23–35
	m.3242G>A	Kidney failure, renal tubular acidosis	Hypertrophic and dilated cardiomyopathy, muscle hypotonia, lactic acidosis	36, 37
tRNA^Phe^	m.616T>C	Tubulointerstitial kidney disease, kidney failure	Severe epilepsy, failure-to-thrive, microcephaly, hypotonia, gastroparesis, growth retardation	38–42
	m.586G>A			
	m.608A>G			
HSP of mtDNA	m.547A>T	Tubulointerstitial kidney disease, kidney failure	No neurological involvement	40
tRNA^Ile^	m.4269A>G	FSGS	Encephalomyopathy, deafness, dilated cardiomyopathy	43
	m.4291T>C	Distal tubular dysfunction, hypomagnesemia	Migraine, sensorineural hearing loss, hypertrophic cardiomyopathy	44
tRNA^Asn^	m.5728A>G	FSGS	Failure to thrive, neurological involvement	45
tRNA^Tyr^	m.5843A>G	FSGS	Dilated cardiomyopathy	46
	m.2905bp deletion	FSGS, necrotizing nephritis, chronic interstitial nephritis	Ataxia, dysarthria, ocular involvement, hearing deficits, seizures	47
*MT-ND5*	m.12425delA	Chronic kidney failure, glomerulocystic disease	Myopathy	48
tRNA^Lys^	m.8344A>G	Tubulopathy	Myoclonic epilepsy and ragged-red fiber disease (MERRF), hearing loss, Kearns−Sayre syndrome, mitochondrial myopathy	49

Asn, Asparagine; DNA, deoxyribonucleic acid; FSGS, focal segmental glomerulosclerosis; HSP, heavy strand promotor; HUPRA, hyperuricemia, pulmonary hypertension, kidney failure and alkalosis; Ile, Isoleucine; Leu, Leucine; Lys, Lysine; MELAS, mitochondrial encephalopathy, lactic acidosis, and stroke-like episodes; mt, mitochondrial; Phe, phenylalanine; Ref, reference; Tyr, tyrosine.

**Table 2:** Genotype phenotype correlation of nuclear DNA mutations involved in mitochondrial cytopathies with kidney involvement

Gene	nDNA mutation	Consequences on protein level	Predominant kidney phenotype	Extrarenal symptoms	Ref
**Respiratory chain assembly and function**					
*BCS1L*	c.830G>A	p.Ser277Asp	Proximal tubulopathy	Hepatic involvement, encephalopathy	50–52
	c.296C>T	p.Pro99Leu			
	c.464C>G, c.1057G>A	p.Arg155Pro, Val353Met			
*SURF1*	c.312del10insAT, c.688C>T	p.Pro104_Leu105insTer, p.Arg230Ter	Tubulopathy	Hypotonia, developmental regression, encephalopathy	53
	c.834G>A	p.Trp278Ter			
	c.312del10insAT, 820–824dupTACAT	p.Pro104_Leu105insTer, out-of-frame duplication			
*COX10*	c.612C>A	p.Asn204Lys	Tubulopathy	Neurological deterioration, leukodystrophy	54
*TMEM70*	c.317-2A>G	Splice site mutation	Renal tubular acidosis, hydronephrosis, kidney failure	Neurological involvement, cardiomyopathy	55, 56
	c.317-2A>G, (c.470T>A, c.628A>C, c.118_119insGT or c.251delC)	Splice site mutation, (ND, p.Thr210pro, p.Ser40CysfsTer11 or ND)			
	c.238C>T	p.Arg80Ter			
	c.316 + 1G>T	Splice site mutation			
	c.336T>A	p.Tyr112Ter			
	c.578_579delCA	p.Thr193Serfs			
	c.535C>T, c.359delC	p.Tyr179His, ND			
*UQCC2*	c.214-3C>G	Splice site mutation	Tubular dysfunction	Severe intrauterine growth retardation, lactic acidosis	57
*ETHE1*	c.505+1G>T	Splice site mutation	Crescentic glomerulonephritis	Ethylmalonic encephalopathy	58
*NDUFAF2*	c.114C>G	p.Tyr38Ter	Renal tubular acidosis	Muscular hypotonia, developmental delay	59
**Mitochondrial protein translation**					
*SARS2*	c.1169A>G	p.Asp390Gly	Tubulopathy, hypomagnesemia, progressive kidney failure	Pulmonary hypertension (HUPRA syndrome)	60, 61
	c.1205G>A	p.Arg402His			
*YARS2*	c.1303A>G	p.Ser435Gly	Tubulopathy	Myopathy, lactic acidosis, anemia	62
*MRPS22*	c.509G>A	p.Arg170His	Tubulopathy	Hypertrophic cardiomyopathy	63
**Post-translational modification of mitochondrial proteins**					
*XPNPEP3*	c.1357G>T	p.Gly453Cys	Kidney failure	Limited to mild neurologic involvement with sensorineural hearing loss and essential tremor	64
	c.931_934delAACA	p.Asn311LfsX5		Mental retardation, seizures, cardiomyopathy	
*DGUOK*	c.165G>A, c.487_490dupGACA	p.Try65Ter, frame shift mutation	Tubulopathy	Developmental delay, hypotonia	65
	c.677A>G, c.592-4_c.592-3delTT	p.His226Arg*,* splice site mutation			
*TSFM*	c.934C>T	p.Arg312Try	Tubulopathy	Hypotonia, liver insufficiency	66
*SUCLA2*	c.534+1G>A	Splice site mutation, multiple exon skipping	Tubulopathy, renal Fanconi syndrome	Progressive hearing loss, epilepsy,	67
*TK2*	c.547C>G, c.760C>T	p.Arg183Gly, p.Arg254Ter	Tubulopathy	CNS and skeletal muscle involvement	68
*COQ2*	Various (Supplementary Table S1)	Various (Supplementary Table S1)	(steroid-resistant) nephrotic syndrome	Neurological, cardiovascular, and ocular involvement, diabetes mellitus	Supplementary Table S1
*COQ6*	Various (Supplementary Table S1)	Various (Supplementary Table S1)	(steroid-resistant) nephrotic syndrome	Sensorineural deafness, occasional neurological involvement	Supplementary Table S1
*COQ8B/ADCK4*	Various (Supplementary Table S1)	Various (Supplementary Table S1)	(steroid-resistant) nephrotic syndrome	Occasional (mild) neurological, ocular, and cardiovascular involvement	Supplementary Table S1
*COQ9*	c.730C>T	p.Arg244Ter	Tubular dysfunction	Neonatal lactic acidosis, seizures, global developmental delay, hypertrophic cardiomyopathy	69, 70

CNS, central nervous system; CoQ_10_, coenzyme Q_10_; DNA, deoxyribonucleic acid; n, nuclear; ND, no data (not reported); Ref, reference.

All mutations are described as pathogenic in the literature.

### Glomerular Diseases

Podocytes are highly differentiated cells with limited regenerative capacity.^84^ The main metabolic pathway for podocytes is anaerobic glycolysis and the fermentation of glucose to lactate.^85^ Therefore, the mechanism of glomerular dysfunction is probably different from that of tubular dysfunction in patients with mitochondrial cytopathies. Brinkkoetter *et al.* hypothesize that a change in podocyte metabolism rather than the loss of mitochondrial oxidative phosphorylation is essential for damage to the podocyte.^85^ Two major mitochondrial cytopathies associated with glomerular dysfunction are due to a m.3243A>G mutation in tRNA^Leu^ gene or to genetic defects in the CoQ_10_ biosynthesis pathway^8^ (Tables 1 and 2, Figure 2).

#### m.3243A>G Mutation in tRNA^Leu^ Gene

The m.3243A>G mutation in the tRNA^Leu^ gene is one of the most common mtDNA point mutations, but the phenotypic expression can be highly variable. This mutation is associated with a wide range of clinical extrarenal manifestations including deafness, diabetes mellitus, neuromuscular involvement, hypertrophic cardiomyopathy, and macular dystrophy.^86^ The m.3243A>G mutation was initially described in mitochondrial myopathy encephalopathy with lactic acidosis and stroke like episodes (MELAS) syndrome, a mitochondrial syndrome manifested in patients who are typically less than 40 years of age.^20^^,^^87^ Low m.3243A>G mutation heteroplasmy levels cause maternally inherited diabetes and deafness (MIDD) syndrome.^88^ The m.3243A>G mutation is associated with focal segmental glomerulosclerosis (FSGS), tubulointerstitial nephritis, and cystic kidney disease (Table 1).^23^ The kidney disease associated with the m.3243A>G mutation generally corresponds to a glomerulopathy with proteinuria, which is below the nephrotic range in two-thirds of patients. The majority of patients are diagnosed with kidney disease in their second or third decade of life, and CKD is present in one-half of these cases.^86^

#### CoQ_10_ Deficiency

**Figure 3:**
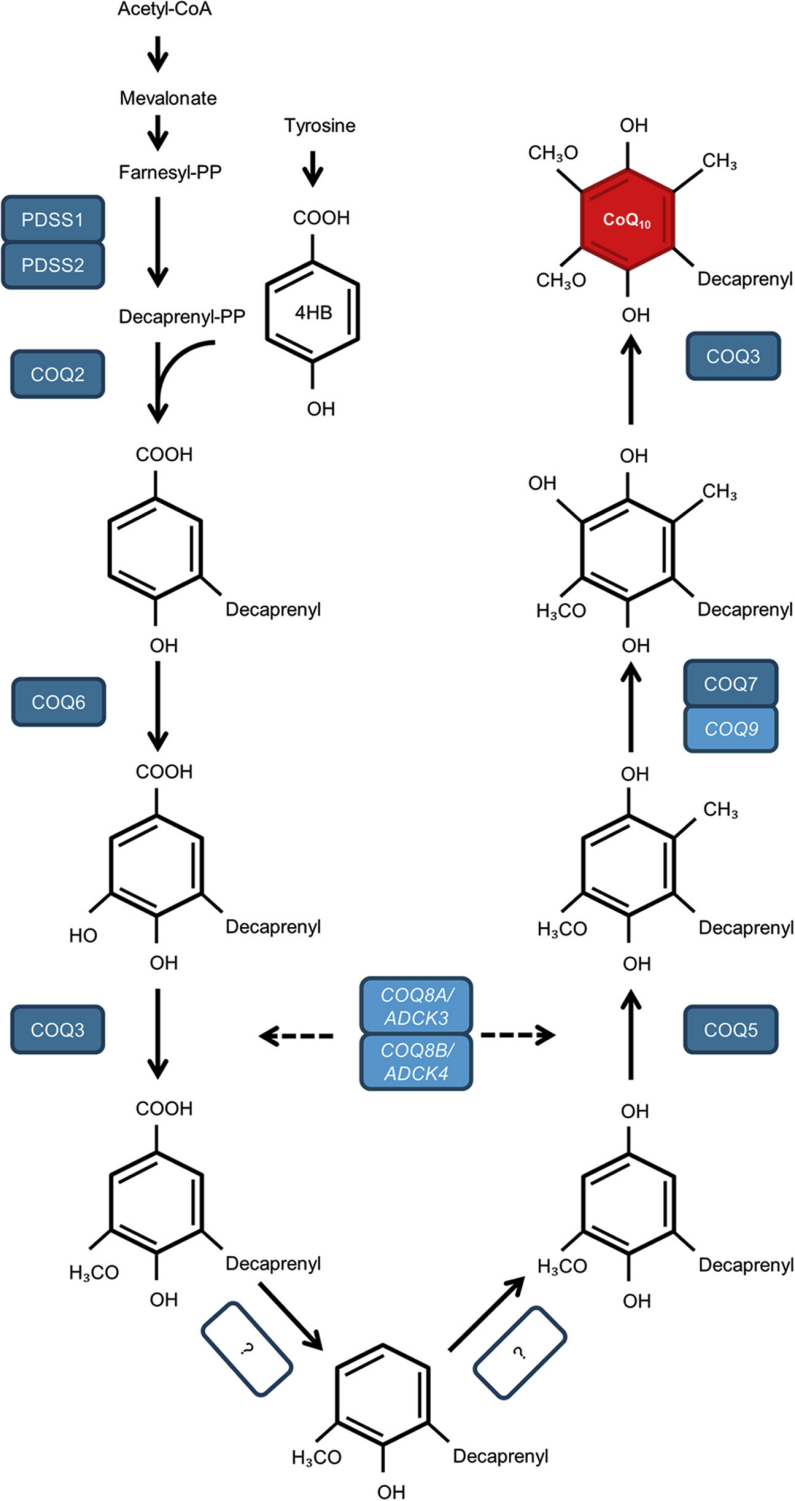
Schematic overview of the CoQ_10_ biosynthesis pathway. At least 15 genes are involved in the CoQ_10_ biosynthesis pathway; however, the biochemical pathway is not yet completely understood. The function of COQ genes shown in italics (COQ8A/ADCK3, COQ8B/ADCK4, and COQ9) has not been fully elucidated. COQ8A/ADCK3 and COQ8B/ADCK4 have a regulatory role,^89^ COQ9 is a lipid-binding protein interacting with COQ7.^91^ The question mark indicates that the enzyme involved in the reaction has not been identified. CoA, coenzyme A; COQ, coenzyme Q gene; PDSS, prenyldiphosphate synthase subunit; PP, pyrophosphate; 4HB, 4-hydroxybenzoate

Primary CoQ_10_ deficiency is a clinically and genetically heterogeneous disorder. Clinical phenotypes range from fatal infantile multisystem disorders to isolated glomerular involvement. In addition, variability exists regarding the age of onset, the different organs involved, and clinical response to CoQ_10_ supplementation. CoQ_10_ is present in the normal diet, but at insufficient levels to supply mitochondria. Therefore, *de novo* biosynthesis in mitochondria is needed, a complex pathway that involves several proteins encoded by *COQ* genes (Figure 3).^16^^,^^89^, ^90^, ^91^ Currently, mutations in 10 different genes involved in the CoQ_10_ pathway have been reported (*PDSS1*, *PDSS2*, *COQ2*, *COQ4*, *COQ5*, *COQ6*, *COQ7*, *COQ8A*/*ADCK3*, *COQ8B*/*ADCK4*, *COQ9*).^92^ A literature search was conducted to identify all reported cases with glomerular involvement to date (February 2020). References of the identified articles were used to search for additional case reports. In total, approximately 200 patients from 130 families with a primary CoQ_10_ deficiency have been described in the literature.^93^ Mutations in *PDSS1*, *PDSS2*, *COQ2*, *COQ6*, and *COQ8B/ADCK4* have been associated with glomerular involvement.^8^ Supplementary Table S1 summarizes clinical and genetic data of all 144 patients with a *PDSS1*, *PDSS2*, *COQ2*, *COQ6*, and *COQ8B/ADCK4* mutation with glomerular involvement reported to date. An overview of the clinical characteristics of these mutations is provided in Table 3. Three cases from our tertiary referral center are described in detail in the Supplementary Case Description, Supplementary Table S2, and Supplementary Figures S1 and S2.

**Table 3:** Overview of clinical characteristics of patients with a *PDSS2*, *COQ2*, *COQ6*, or *COQ8B*/*ADCK4* mutation and kidney involvement reported in literature

Clinical characteristics	*PDSS2* gene	*COQ2* gene	*COQ6* gene	*COQ8B/ADCK4* gene
Patients reported, n Families reported, n	7 patients 5 families	28 patients 23 families	26 patients 19 families	82 patients 47 families
Median age at onset (range)	6 mo (birth to 3 yr)	9 mo (birth to 18 yr)	2.5 yr (2 mo to 16 yr)	12 yr (10 days to 32 yr)
Reported extrarenal symptoms, % of patients (proportion)	100% (6/6)	68% (17/25)	90% (20/22) Sensorineural deafness 81% (18/22)	40% (26/65)
Kidney failure, % of patients (proportion)	100% (3/3)	63% (12/19)	72% (16/22)	73% (54/74)
Median age at kidney failure (range)	8 yr (6 mo to 9 yr)	2.1 yr (3 wk to 19 yr)	3.2 yr (5 mo to 9 yr)	14.9 yr (6 to 35 yr)
Time to kidney failure (range)	6 yr (2 wk to 7 yr)	5 mo (0 to 2.5 yr)	1 yr (1 mo to 3 yr)	1 yr (0 to 13 yr)
Histopathology, % of patients (proportion)				
Glomerular/diffuse mesangial sclerosis	100% (3/3)		5% (1/21)	
(c)FSGS		69% (11/16)	86% (18/21)	98% (43/44)
MPGN			10% (2/21)	
Nephrocalcinosis				13% (6/45)
CoQ_10_ supplementation				
% of Patients	80% (4/5)	65% (15/23)	65% (15/23)	37.5% (30/82)
Dosing range	5−20 mg/kg per day	5−60 mg/kg per day	20−100 mg/kg per day	3.3−30 mg/kg per day
Treatment effect, % of patients (proportion)				
No effect	50% (2/4)			
Improvement clinical condition	50% (2/4)			
Improvement neurological condition		14% (2/14)		
Neurological deterioration		29% (4/14)		
Improvement kidney symptoms		43% (6/14)	56% (5/9)	43% (13/30)
No effect on kidney symptoms		29% (4/14)		57% (17/30)
Improvement sensorineural deafness			83% (5/6)	
No effect on sensorineural deafness			17% (1/6)	

*ADCK4*, AarF Domain Containing Kinase-4; *COQ2*, Coenzyme Q2; *COQ6*, Coenzyme Q6; *COQ8B*, Coenzyme Q8B; (c)FSGS, (collapsing) focal segmental glomerulosclerosis; MPGN, membranoproliferative glomerulonephritis; *PDSS2*, decaprenyl-diphosphate synthase subunit 2.

Proportion in parenthesis represents the number of patients with the characteristic/total number of patients for whom this characteristic was reported in literature.

Only 1 patient with a *PDSS1* mutation and glomerular involvement was reported in literature and is therefore not included in Table 3. This patient is reported in Supplementary Table S1. Three patients from our clinic are described in detail in the Supplementary File, including Supplementary Case Description, Supplementary Table S2, and Supplementary Figures S1 and S2.

PDSS1 and PDSS2 encode a subunit of the enzyme required for synthesis of the decaprenyl tail of CoQ_10_ (Figure 3). Only 1 patient with a *PDSS1* mutation and glomerular involvement has been reported in literature.^94^ The patient presented before the age of 6 months with nephrotic syndrome, failure to thrive, and developmental delay. She rapidly developed kidney failure and died at the age of 16 months. To date, *PDSS2* mutations have been identified in 7 patients with glomerular mitochondrial cytopathies associated with CoQ_10_ deficiency from 5 unrelated families. Gasser *et al.* showed that a *PDSS2* haplotype was associated with a significantly increased risk of FSGS and collapsing glomerulopathy in European American patients.^95^ Although the number of reported patients with a *PDSS2* mutation is limited, the patients generally presented at a young age and rapidly developed kidney failure. Furthermore, severe extrarenal involvement was reported in these patients, resulting in a poor prognosis. Saiki *et al.* showed that CoQ_10_ supplementation rescued proteinuria and interstitial nephritis in PDSS2-deficient mice.^96^ In clinical practice, CoQ_10_ supplementation (5−20 mg/kg per day) was started in 4 patients, and 2 of them showed improvement in their clinical condition.^97^

*COQ2* encodes the enzyme para-hydroxybenzoate-polyprenyl-transferase required for the second step of the final pathway of CoQ_10_ biosynthesis (Figure 3).^98^ Steroid-resistant nephrotic syndrome (SRNS) usually develops within the first 2 years of life and often represents the first symptom of the disease, with or without neurologic symptoms. As shown in Table 3, 12 of 19 patients showed a rapid decline in kidney function. Furthermore, 17 of 25 patients showed some degree of extrarenal involvement. Patients who presented with decreased kidney function did not show improvement of kidney function after CoQ_10_ supplementation. In patients presenting with nephrotic syndrome, a decrease in proteinuria may occur. Patients with severe neurological involvement showed neurological deterioration irrespectively of treatment with CoQ_10_, even when started immediately after birth (Supplementary Table S1). As expected, no recurrence of proteinuria occurred after kidney transplantation. In contrast to other genes involved in the CoQ_10_ biosynthesis, there is a genotype−phenotype correlation for mutations in the *COQ2* gene.^99^ Mutations in the *COQ2* gene have been associated with a wide spectrum of phenotypes, including a rapidly fatal, neonatal onset, multisystemic disease^100^; SRNS, which can be associated with encephalopathy; and late-onset encephalopathy with retinopathy mimicking multiple system atrophy.^8^^,^^99^^,^^101^^,^^102^ As previously described by Desbats *et al.*, patients with severe phenotypes harbored 2 alleles that significantly impaired CoQ_10_ production.^99^ In contrast, patients harboring a compound heterozygous *COQ2* mutation showed a milder clinical phenotype, including a higher age of onset, less severe clinical symptoms, and absence of extrarenal manifestations.

The enzyme CoQ_10_ monooxygenase 6 (COQ6) is required for biosynthesis of CoQ_10_ and catalyzes the C5 hydroxylation step of the quinine ring (Figure 3). Ozeir *et al.* showed that Coq6 is not required for the C1-hydroxylation.^103^ As shown in Table 3, most of the affected children presented with SRNS and sensorineural deafness, generally at older ages than those reported for patients with *COQ2* mutations.^104^ The effect of CoQ_10_ supplementation was not easy to evaluate, as most reported patients rapidly developed kidney failure. However, patients who did not rapidly develop kidney failure showed improvement of proteinuria (Table 3, Supplementary Table S1). In contrast, CoQ_10_ supplementation did not improve sensorineural deafness in most patients.

COQ8B/ADCK4 (AarF Domain Containing Kinase-4) interacts with components of the CoQ_10_ biosynthesis pathway (Figure 3).^105^, ^106^, ^107^ COQ8B/ADCK4 is expressed in podocytes and localized to mitochondria and foot processes.^106^ A total of 82 patients with a *COQ8B/ADCK4* mutation and kidney involvement have been reported to date (Table 3). Symptoms typically present in puberty, and CKD was diagnosed at presentation in one-fourth of the reported patients. Moreover, 54 of 74 patients (73%) developed kidney failure, with a median time to kidney failure of 1 year. In most patients, a kidney biopsy showed FSGS. Interestingly, Park *et al.* reported accompanying medullary nephrocalcinosis in 6 Korean patients.^108^ Treatment with CoQ_10_ was started in less than 50% of the reported patients. In 17 of 30 patients (57%), no improvement of kidney function was seen, especially in patients who already presented with impaired kidney function. However, if started early, patients did show a decrease in proteinuria (Table 3).

### CoQ_10_ IN DEPTH

Primary CoQ_10_ deficiency is a clinically and genetically highly variable disorder, as illustrated by the patients reported in Supplementary Table S1. Early diagnosis is crucial. as oral supplementation of CoQ_10_ can limit disease progression and improve clinical symptoms.

### Diagnostic Methods and Monitoring

The diagnosis of primary CoQ_10_ deficiency is established with the identification of pathogenic variants in any of the genes encoding for the proteins directly involved in CoQ_10_ biosynthesis (Figure 3). Nowadays, genetic testing using next-generation sequencing is substantially less time intensive than a few years ago; however, because treatment should be started as early as possible, additional diagnostic tests are still indispensable to enable rapid identification of CoQ_10_ deficiency. Classic biochemical analyses remain important in the diagnostics of mitochondrial cytopathies. Specifically for CoQ_10_ deficiency, analysis of the combined activity of complex I to III and/or II and III is important. Furthermore, initial diagnostic testing should include measurement of blood lactate and alanine (as a measure of impaired energy production in skeletal muscle and long-standing pyruvate accumulation, respectively), even though normal levels do not exclude CoQ_10_ deficiency.^109^^,^^110^ In addition, assessment of CoQ_10_ levels may be helpful in diagnosis and follow-up. Measurement of CoQ_10_ in skeletal muscle is considered the gold standard test for diagnosing CoQ_10_ deficiency; however, with the availability of relatively rapid genetic diagnostics, muscle biopsies are not routinely performed anymore.^109^^,^^111^ Measurement of CoQ_10_ levels in skin fibroblasts may be another option in patients with a primary CoQ_10_ deficiency.^90^ Nevertheless, a less invasive screening test is desirable. CoQ_10_ levels can be measured in different biological specimens.^93^^,^^112^, ^113^, ^114^, ^115^, ^116^ CoQ_10_ levels in plasma appear to be highly dependent on dietary intake and are therefore not reflective of the levels in tissues.^117^ Moreover, CoQ_10_ levels in peripheral cells do not seem to accurately reflect the amount of CoQ_10_ in tissues, and the exact value of other biological specimens in the diagnostics of CoQ_10_ deficiency remains to be elucidated.

### Pathophysiology

The pathogenesis of CoQ_10_ deficiency involves different aspects. Interestingly, variable degrees of CoQ_10_ deficiency appear to cause different defects of ATP synthesis and oxidative stress. Quinzii *et al.* show that severe CoQ_10_ deficiency (<20% of normal) results in significant bioenergetic defects without oxidative stress. In contrast, intermediate CoQ_10_ deficiency (30%−40% of normal) causes moderate bioenergetic defects but a significant increase in ROS production, indicating that residual activity of the respiratory chain is indispensable to produce ROS.^118^ Podocytes are known to be susceptible to oxidative damage,^119^ whereas impaired mitochondrial biogenesis did not result in a developmental or pathological change in podocytes.^85^ This supports the concept that podocytes are independent of mitochondrial ATP production but susceptible to oxidation. In line with this, Desbats *et al.* show that patients with a severe presentation of CoQ_10_ deficiency (presentation at birth with multi-organ failure) due to a *COQ2* mutation harbored 2 alleles that markedly impaired CoQ_10_ production. In contrast, patients with a milder phenotype including isolated nephrotic syndrome had at least 1 allele that allowed significant residual CoQ_10_ biosynthesis for ROS production.^99^ Zhu *et al.* used a Drosophila model to investigate COQ2 nephropathy. Silencing coq2 led to abnormal localization of slit diaphragms, collapse of lacunar channels, and dysmorphic mitochondria. Furthermore, increased levels of ROS were found in this model, and dietary supplementation with CoQ_10_ partially rescued these defects.^120^ Mutations in *COQ8B/ADCK4* account for the highest number of patients with kidney disease secondary to CoQ_10_ deficiency (Supplementary Table S1). Ashraf *et al.* showed that knockdown of *COQ8B/ADCK4* in zebrafish resulted in the characteristic triad of nephrotic syndrome.^106^ Moreover, *COQ8B/ADCK4* is required for CoQ_10_ biosynthesis and mitochondrial function in podocytes.^121^ Compared to other CoQ_10_ biosynthesis defects, mutations in *COQ8B/ADCK4* seem to result in a less severe clinical entity, with a more prominent kidney phenotype, higher age at onset of SRNS, and good patient survival owing to the lack of extrarenal manifestations. The selective glomerular phenotype of patients with *COQ8B/ADCK4* mutations may be the result of relative enrichment of COQ8B/ADCK4 and lacking expression of the related protein COQ8A/ADCK3 in podocytes, whereas COQ8A/ADCK3 expression exceeds that of COQ8A/ADCK4 in most other body tissues.^106^ Ashraf *et al.* showed that knockdown of *COQ8B/ADCK4* in podocytes reduced their migration phenotype, which could be reversed by the addition of CoQ_10_.^106^ The relatively mild phenotype observed in patients with *COQ8B/ADCK4* defects is probably related to the fact that the encoded enzyme has a modulatory function without catalytic activity, enabling residual CoQ_10_ synthesis even in the complete absence of this protein.^8^ Moreover, other genes may partially compensate for the absence of COQ8A*/*ADCK4.^122^

### Treatment

Oral administration of CoQ_10_ is the treatment strategy for affected individuals with a CoQ_10_ deficiency. As was previously shown by Montini *et al.* and various other clinical reports, CoQ_10_ can block the progression of the disease.^12^ Although severe neurological and kidney damage cannot be reversed, treatment may also be initiated to prevent development of additional extrarenal symptoms. Different formulations of CoQ_10_ are available, including an oxidized form (ubiquinone), a reduced form (ubiquinol), and analogs such as idebenone. Recently, Kleiner *et al.* proposed that CoQ_10_ supplementation causes an increase in CoQ_10_ levels, rescuing sulfide oxidation and thereby preventing kidney failure.^19^ Unfortunately, clinical studies regarding efficacy are lacking,^92^ and the optimal dose and form of oral CoQ_10_ are still under debate. In the literature, the daily dose of CoQ_10_ ranges from 5 to 100 mg/kg (Supplementary Table S1), with most clinicians describing a dose between 30 and 50 mg/kg per day. Atmaca *et al.* recently published long-term follow-up results of CoQ_10_ supplementation in patients with a *COQ8B/ADCK4* mutation, and observed maximum reduction of proteinuria at 6 months of treatment.^123^ After a median follow-up duration of 25.3 months following CoQ_10_ administration (20−30 mg/kg per day), proteinuria was significantly decreased, whereas kidney function was preserved.^123^^,^^124^ Of note, 4 of 8 reported patients received angiotensin-converting enzyme inhibitors or an angiotensin receptor blocker in addition to CoQ_10_, which may have contributed to the decrease in proteinuria. For patients with mutations in the *COQ2* gene, however, results were less favorable. Eroglu *et al.* reported 4 patients of 2 different families, in which 2 patients were started on CoQ_10_ supplementation immediately after birth or at first presentation.^125^ Despite early treatment and an initial good response in terms of nephrotic syndrome and hyperglycemia, the patients still developed severe neurological problems and eventually died at 31 and 14 months of age, respectively.^125^

Interestingly, 3 patients with a *COQ6* mutation reported in the literature and 1 of the patients with a homozygous pathogenic *COQ2* mutation from our clinic (Supplementary File) showed a response to cyclosporine therapy with regard to the nephrotic syndrome.^126^ CoQ_10_ deficiency can directly lead to the opening of the mitochondrial permeability transition pore (MPTP). Moreover, an increased amount of ROS can also induce mitochondrial permeability transition.^127^ Cyclosporine has the capacity to inhibit MPTP through interaction with cyclophilin D, an essential component of the MPTP, and thereby reduce permeability.^128^ In line with this, Heeringa *et al.* showed that incubation of *COQ6* knockdown podocytes with cyclosporine had a mild rescue effect.^104^ Our additional hypothesis is that the increase in ROS due to CoQ_10_ deficiency may have an impact on the cytoskeleton of the podocytes. Cyclosporine is known for its podocyte-stabilizing capacities and might therefore cause a partial response in these patients.^129^

A new potential therapeutic approach was recently reported for mitochondrial dysfunction due to CoQ_10_ deficiency. The treatment of Coq6 knockout mice with 2,4-dihydroxybenzoic acid (4-diHB), an analog of a CoQ precursor molecule, prevented kidney dysfunction and reversed podocyte migration rate impairment.^130^ A potential role for 2,4diHB was suggested for the treatment of CoQ10 deficiency caused by *COQ8B/ADCK4* mutations as well.^121^ Moreover, Ozeir *et al.* demonstrated that analogs of 4-HB can bypass a deficient CoQ biosynthetic enzyme.^103^ In addition, hydroxylated analogs of 4-HB, 3,4-dihydroxybenzoic acid and/or vanillic acid were marked as a potential therapeutic intervention for CoQ_10_ deficiency due to a *COQ6* mutation.^131^^,^^132^

Currently, no curative treatment options for mitochondrial cytopathies are available, besides CoQ_10_ supplementation for patients with a CoQ_10_ deficiency. Typically, patients receive supportive treatment, and other treatment options involve dietary supplements, vitamins, and antioxidants.^133^ A 2012 Cochrane review of mitochondrial therapies has found little evidence supporting the use of any vitamin or cofactor intervention.^134^ Furthermore, there is early evidence for a therapeutic role of L-arginine and citrulline therapy for MELAS-related strokes. The evidence supporting the use of CoQ_10_ in mitochondrial diseases other than primary CoQ_10_ is sparse.^133^

## Conclusion

In this review, an overview of kidney involvement in genetic mitochondrial cytopathies is provided. Kidney manifestations reported in the setting of mitochondrial dysfunction include tubular dysfunction, interstitial nephritis, glomerular pathology, and, in rare cases, cystic disease. Special focus on CoQ_10_ deficiency was presented to emphasize early diagnosis of primary CoQ_10_ deficiency, as oral supplementation of CoQ_10_ may limit disease progression and improve clinical symptoms. A combination of genetic and metabolic diagnostics, including targeted whole-exome sequencing and blood lactate and alanine levels at presentation, should be performed in children with nephrotic syndrome presenting in adolescence or at a young age (<3 years) and in patients with nephrotic syndrome at any age with extrarenal symptoms. In case none of these criteria is present, we recommend awaiting steroid responsiveness for 4 weeks. In case patients do not respond to steroids after 4 weeks of treatment, we would advise performing genetic diagnostics. Moreover, starting CoQ_10_ treatment should be considered in expectation of the final genetic diagnosis. Lifelong CoQ_10_ supplementation should be considered, as sustained remission of nephrotic syndrome has been described and extrarenal symptoms may be prevented. Finally, a multidisciplinary approach is required, given the number of organs that can be involved in patients suffering from a mitochondrial cytopathy.

## Disclosure

All the authors declared no competing interests.
