# Supplementary material for: Mitochondrial Disease and the Kidney With a Special Focus on CoQ10 Deficiency
Source: Kidney Int Rep. 2020 Oct 10;5(12):2146–59. doi: 10.1016/j.ekir.2020.09.044 (PMC7710892; doi:10.1016/j.ekir.2020.09.044)
Supplement: Supplementary File (PDF) [file mmc1.pdf]

Table of contents

1. Supplemental Table 1 Overview of patients reported in literature with a *PDSS1*, *PDSS2*, *COQ2*, *COQ6*, or *COQ8B*/*ADCK4* mutation and glomerular involvement.
2. Detailed description of three patients
3. Supplemental Table 2 Clinical characteristics of our 3 patients with a primary CoQ_{10} deficiency
4. Supplemental Figure 1a Light microscopy image patient 2
   Supplemental Figure 1b Electron microscopy patient 2
   Supplemental Figure 1c Electron microscopy patient 3
   Supplemental Figure 2 Disease course of patient 3
5. Supplemental references

Supplemental Table 1: Overview of patients reported in literature with a *PDSS1*, *PDSS2*, *COQ2*, *COQ6*, or *COQ8B*/*ADCK4* mutation and glomerular involvement.

PDSS1 gene

	**Nucleotide alterations**	**Consequences at protein level**	**Hom/ Het**	**Exon**	**Age at onset (y)**	**Gender**	**Consanguinity**	**Family history**	**Presenting symptoms**	**Extrarenal symptoms**	**Age at kidney failure (y)**	**CoQ_{10} supplementation**	**Dose**	**Treatment effect**	**Histopathology**	**Outcome**	**Ref**
1	c.661_662insT<br>c.1108A>C	p.Arg221Ter<br>p.Ser370Arg	Het<br>Het	7<br>12	<0.5	F	ND	ND	Nephrotic syndrome	Developmental delay, failure to thrive	Yes, age ND	No	NA	NA	Acute tubular epithelial damage	Died at 16 months of age, **kidney failure**	S1

PDSS2 gene

	**Nucleotide alterations**	**Consequences at protein level**	**Hom/ Het**	**Exon**	**Age at onset (y)**	**Gender**	**Consanguinity**	**Family history**	**Presenting symptoms**	**Extrarenal symptoms**	**Age at kidney failure (y)**	**CoQ_{10} supplementation**	**Dose**	**Treatment effect**	**Histopathology**	**Outcome**	**Ref**
1	c.964C>T<br>c.1145C>T	p.Gln322Ter<br>p.Ser382Leu	Het<br>Het	6<br>8	At birth	M	No	Healthy sister, healthy parents	Neonatal pneumonia, hypotonia<br>7 m: nephrotic syndrome	3 m: seizures cortical blindness	ND	Yes	50 mg/day at age of 3 m	No effect	ND	Died at 8 months of age	S2
2	c.485A>G<br>c.1042_1148-2816 del	p.His162Arg, 107 base long deletion	Het<br>Het	3<br>8	0.6	M	No	Healthy parents, no siblings	Nephrotic syndrome	Encephalomyopathy, hypertrophic cardiomyopathy, clinically suspected deafness and retinitis pigmentosa	0.625	Yes	20 mg/kg/ day	No effect	Diffuse mesangial sclerosis	Died at 8 months of age, kidney failure	S3
3	c.1151C>A	p.Ala384Asp	Hom	8	At birth	M	No	ND	SRNS	ND	ND	ND	ND	ND	ND	ND	S4

4	c.1145C>T	p.Ser382Leu	Hom	8	1.9	M	Yes	ND	SRNS	Cerebral palsy, intellectual disability	ND	ND	ND	ND	ND	ND	S4
5-I	ND*	ND	ND	ND	3	M	ND	Healthy parents, healthy brother, two affected sisters (5-II, 5-III)	Nephrotic syndrome	Retinitis pigmentosa, optic nerve atrophy, cataract, deafness, hypertrophic cardiomyopathy, progressive ataxia, dystonia,	9	Oral ubidecarenone	5 mg/kg/day	Improvement clinical condition	ND	kidney failure, kTx	S5
5-II	ND*	ND	ND	ND	0.5	F	ND	Healthy parents, healthy brother, one affected brother and sister (5-I, 5-III)	Nephrotic syndrome	Sensorineural deafness, visual loss, delayed motor development	ND	No	NA	ND	Glomerular sclerosis	Died at 8 y of age after rapid neurological deterioration	S5
5-III	ND*	ND	ND	ND	1	F	ND	Healthy parents, healthy brother, one affected brother and sister (5-I, 5-II)	Nephrotic syndrome	Sensorineural deafness, nystagmus, myopia, moderate ataxia, moderately mentally disabled	8	Oral ubidecarenone	5 mg/kg/day	Improvement clinical condition	Glomerular sclerosis	kidney failure, kTx	S5

* Reported by Rahman et al. as personal communication.[6]

COQ2 gene

	**Nucleotide alterations**	**Consequences at protein level**	**Hom /Het**	**Exon**	**Age at onset (y)**	**Gender**	**Consanguinity**	**Family history**	**Presenting symptoms**	**Extrarenal symptoms**	**Age at kidney failure (y)**	**CoQ_{10} supplementation**	**Dose**	**Treatment effect**	**Histopathology**	**Outcome**	**Ref**
1-I	c.890A>G	p.Tyr297Cys	Hom	5	1	M	Yes	Affected sister (1-II)	SRNS	Hypotonia, mild psychomotor delay, optic nerve atrophy, rod-cone retinopathy, status epilepticus	1.5	Yes	30 mg/kg/day	Improvement of neurological manifestations, no improvement of kidney function.	FSGS	kidney failure, PD, successful transplant age of 3y.	S7,S8

1-II	c.890A>G	p.Tyr297Cys	Hom	5	0.75	F	Yes	Affected brother (1-I)	Nephrotic syndrome	None	NA (duration fu ND)	Yes	30 mg/kg/day	ND	FSGS	Normal kidney function	S7,S8
2	c.1198delT	p.Asn401fs*415	Hom	7	2d	F	No (same geographical region and same patronymic)	Healthy parents	Nephrotic syndrome and liver failure	Neurological distress at birth, liver failure, anemia, pancytopenia, diabetes mellitus, seizures	ND	No	NA	NA	Not performed	Died at 12 days of age, multiorgan failure	S9
3	c.437G>A<br>c.1159C>T §	p.Ser146Asn<br>p.Arg387Ter	Het<br>Het	2<br>7	At birth	F	ND	ND	Cardiomyopathy	Hypertrophic cardiomyopathy, prominent occiput, 11 ribs, spade-shaped hands, externally rotated flat feet, an anteriorly placed anus, diabetes mellitus	ND	No	NA	NA	Autopsy: chronic renal tubular disease suggestive of smoldering tubular necrosis	Died at 2 months of age, multi organ failure	S10
4	c.590G>A<br>c.683A>G	p.Arg197His<br>p.Asn228Ser	Het<br>Het	3<br>3	1.5	M	No	Healthy parents, healthy brother	SRNS	None	1.7	Yes	30 mg/kg/day	Normal neurologic examination	Collapsing glomerulopathy, numerous dysmorphic mitochondria	Nephrectomy, PD	S11
5	c.437G>A	p.Ser146Asn	Hom	2	5d	M	Distantly related	Healthy parents	Acute kidney failure	Epileptic encephalopathy, hypotonia	3 w	No	NA	NA	Severe crescentic glomerulonephritis, numerous dysmorphic mitochondria	Died at 6 months of age	S11
6-I	c.905C>T	p.Ala302Val	Hom	5	At birth	F	Yes	Healthy parents, twin brother (6-II) affected	Respiratory deterioration	Premature birth (29+5), feeding problems, generalized edema (without proteinuria), seizures, apneas, generalized hypotonia, dystonic	NA	No	NA	NA	Not performed	Died at 5 months of age	S12

										hyperkinetic movements							
6-II	c.905C>T	p.Ala302Val	Hom	5	At birth	M	Yes	Healthy parents, twin sister (6-II) affected	Respiratory deterioration	Premature birth (29+5), feeding problems, generalized edema (without proteinuria), seizures, apneas, generalized hypotonia, dystonic hyperkinetic movements	NA	No	NA	NA	Not performed	Died 6 months of age	S12
7	c.701delT<br>c.683A>G	p.Leu234fs*14<br>p.Asn228Ser	Het<br>Het	4<br>ND	2	F	ND	ND	SRNS	None	3	ND	ND	ND	ND	kidney failure after 6 months, PD	S13
8	c.326G>A	p.Ser109Asn	Hom	2	3w	M	No	Healthy parents	Myoclonic seizures and hypertrophic cardiomyopathy.<br>Nephrotic syndrome (at age of 4 months)	Myoclonic seizures and hypertrophic cardiomyopathy, nystagmoid movements, epilepsia partialis continua, no visual pursuit.	0.4	Yes	5-30 mg/kg/day	No clinical improvement	FSGS, slight pleomorphic mitochondria	Died at 5 months of age	S14
9	c.545T>G	p.Met182Arg	Hom	2	At birth	F	Yes	Unaffected brother, sister heterozygous carrier	Severe lactic acidosis, proteinuria, dicarboxylic aciduria, hepatic insufficiency	Severe lactic acidosis, hepatic insufficiency, dilation of left ventricle	ND	No	NA	NA	Not performed	Died after 23 hours due to rapid neurological deterioration	S15

10	c.1159C>T<br>c.973A>G	p.Arg387Ter<br>p.Thr325Ala	Het<br>Het	7<br>6	0.75	M	ND	ND	Nephrotic syndrome	None	NA (fu age 2.4 yrs)	Yes	30-50 mg/kg/ day	Increase in leukocyte CoQ_{10} level. Decreased proteinuria and improved serum albumin levels.	Focal mesangial sclerosis and collapsing glomerulopathy. Few podocytes with increased numbers of mitochondria, some dysmorphic.	Stable kidney function.	S16
11	c.683A>G<br>c.881C>T	p.Asn228Ser<br>p.Thr294Ile	Het<br>Het	3<br>5	10	M	NA	ND	SRNS	None	11	Yes	30 mg/kg/ day	Increase in leukocyte CoQ_{10} level, no clinical improvement. No recurrence of proteinuria after kTx.	FSGS<br>Few podocytes with increased numbers of mitochondria, some with dysmorphic features.	kidney failure, HD, kTx	S16
12	c.683A>G<br>c.176dupT	p.Asn228Ser<br>p.Ala60Argfs *33	Het<br>Het	3<br>1	2	M	ND	ND	SRNS	None	NA (fu age 4yrs, fu 20 months)	Yes	30-50 mg/kg/ day	Decrease in proteinuria.	FSGS<br>Podocyte mitochondria were plentiful and occasionally dysmorphic	Normal serum albumin	S16
13	c.518G>A<br>c.973A>G	p.Arg173His<br>p.Thr325Ala	Het<br>Het	2<br>6	0.9	M	No	Healthy parents, two unaffecte d sisters, one older sister died of nephropa thy at one year of age	SRNS	Mild motor development retardation and moderate language retardation	NA (fu age 1.2 yrs, fu 3 months)	Yes	30 mg/kg/ day	Decreased proteinuria and improved serum albumin levels, improved motor development.	ND	Normal kidney function	S17
14-I	c.1169G>C	p.Gly390Ala	Hom	7	18	F	Prob ably a com mon ance stor	Cousin (14-II) affected.	SRNS	Juvenile myoclonic epilepsy	19	Yes	ND	No recurrence of proteinuria, no neurological symptoms.	FSGS	kidney failure, kTx	S18
14-II	c.1169G>C	p.Gly390Ala	Hom	7	16	F	Prob ably a	Parents and younger brother	SRNS	Headache associated with phono-	18	Yes	ND	No recurrence of proteinuria, no neurological symptoms.	FSGS	kidney failure, kTx	S18

							common ancestor	with normal kidney function. Cousin (14-I affected)		and photophobia							
15-I	c.437G>A	P.Ser146Asn	Hom	2	4d	M	ND	Affected sister (15-II)	Neonatal diabetes, proteinuria	Refractory seizures, severe encephalopathy, neonatal diabetes, increased liver enzymes	ND	Yes	30 mg /kg/day (started at three months of age)	Improvement of proteinuria. Neurological deterioration	LM unremarkable changes EM Numerous dysmorphic mitochondria	Neurological deterioration. Died at 4.5 months of age.	S19
15-II	c.437G>A	P.Ser146Asn	Hom	2	At birth	F	ND	Affected brother (15-I)	Neonatal diabetes, proteinuria	Focal clonic seizures, encephalopathy, neonatal diabetes	2.5	Yes	30 mg/kg/day started immediately after birth	Improvement of proteinuria. Neurological deterioration	ND	Died of multi organ failure at 31 months of age	S19
16-I	c.437G>A	P.Ser146Asn	Hom	2	0.25	F	ND	Affected brother (16-II)	Nephrotic syndrome	Refractory seizures	0.25	No	NA	NA	Fetal glomerular appearance and visceral epithelial hypertrophy	Died at six months of age due to respiratory distress and multi organ failure	S19
16-II	c.437G>A	p.Ser146Asn	Hom	2	5 d	M	ND	Affected sister (16-I)	Poor feeding Lab: hyponatremia, proteinuria, hyperglycemia	Neonatal diabetes, focal clonic seizures	ND	Yes	30-60mg/kg/day start at day 5	Neurological deterioration.	ND	Died at 14 months of age.	S19
17	c.683A>G	p.Asn228Ser	Hom	3	ND	M	ND	ND	SRNS	ND	ND	ND	ND	ND	ND	ND	S4
18	c.518G>A<br>c.683A>G	p.Arg173His<br>p.Asn228Ser	Het<br>Het	3<br>3	2.5	M	No	ND	SRNS	ND	ND	ND	ND	ND	ND	Remission CsA treatment	S4
19	c.683A>G<br>c.856C>T	p.Asn228Ser<br>p.Leu286Phe	Het<br>Het	3<br>5	1.25	F	No	ND	SRNS	ND	ND	ND	ND	ND	ND	SR, partial remission CsA	S4
20	c.890T>C	p.Tyr297Cys	Hom	5	0.4	M	Yes	ND	SRNS	microcephalus	ND	ND	ND	ND	ND	ND	S4
21	c.832T>C §§	p.Cys278Arg	Hom	ND	0.5	F	Yes	1 healthy sister (heterozygous mutation carrier), 1 sister with mucopolysacchari	SRNS	None	0.5 (1 week after diagnosis)	No	NA	NA	Extensive fusion of epithelial podocytes, degeneration of podocyte vacuoles, tubular-interstitial lesion	kidney failure, died a few weeks after diagnosis	S35

								des (died at age of 11y), 1 sister with NS (died at age of 8m)									
22	c.590G>A<br>c.683A>G	P.Arg197His<br>P.Asn228Ser	Het<br>Het	3<br>3	2.5	F	No	1 healthy brother	Multi drug resistant nephrotic syndrome	None	2.5 (6 weeks after diagnosis)	Yes	30 mg/kg/day	No recurrence proteinuria after kTx, no extrarenal involvement.	cFSGS	Bilateral nephrectomy due to fluid retention, kTx, no recurrence FSGS	-
23	c.683A>G	P.Asn228Ser	Hom	3	1.8	F	Yes	1 healthy sister	SRNS	Decreased muscle tone, poor reflexes	NA (fu age 5.5 yrs)	Yes	20-30mg/kg/day	No extrarenal involvement.	cFSGS	In remission	-

COQ6 gene

	Nucleotide alterations	Consequences at protein level	Hom /Het	Exon	Age at onset (y)	Gender	Consanguinity	Family history	Presenting symptoms	Extrarenal symptoms	Age at kidney failure (y)	CoQ_{10} supplementation	Dose	Treatment effect	Histopathology	Outcome	Ref
1-I	c.763G>A	p.Gly255Arg	Hom	7	6.4	ND	Yes	3 affected siblings (1-II, 1-III, 1-IV)	SRNS	Congenital sensorineural deafness	9.3	No	NA	NA	FSGS	kidney failure	S20
1-II	c.763G>A	p.Gly255Arg	Hom	7	0.3	ND	Yes	3 affected siblings (1-I, 1-III, 1-IV)	SRNS	Congenital sensorineural deafness	1.7	No	NA	NA	ND	Died at 17.5 y of age	S20
1-III	c.763G>A	p.Gly255Arg	Hom	7	1.2	ND	Yes	3 affected siblings (1, I, 1-II, 1-IV)	SRNS	Sensorineural deafness, ataxia	1.4	No	NA	NA	FSGS	Died at 6.5 y of age	S20
1-IV	ND	ND	ND	ND	<1.0	ND	Yes	3 affected siblings (1-II, 1-II, I-III)	SRNS, cyclophosphamide resistant	Congenital sensorineural deafness	3.0	No	NA	NA	FSGS	Diet at 5.0 y of age	S20
2-I	c.763G>A	p.Gly255Arg	Hom	7	0.3	ND	Yes	2 affected siblings (2-II, 2-III)	SRNS	Seizures	0.4	No	NA	NA	Diffuse mesangial sclerosis	Died (age ND)	S20

2-II	c.763G>A	p.Gly255Arg	Hom	7	0.3	F	Yes	2 affected siblings (2-I, 2-III)	SRNS	Sensorineural deafness, facial dysmorphism	0.4	Yes	100mg/day	Improvement of sensorineural deafness	ND	kidney failure	S20
2-III	c.763G>A	p.Gly255Arg	Hom	7	0.16	ND	Yes	2 affected siblings (2-I, 2-II)	Proteinuria	Sensorineural deafness, bilateral nephrolithiasis, growth retardation	NA (fu 15 months)	Yes	30mg/kg/day	No effect on sensorineural deafness, decrease in proteinuria	ND	Normal kidney function	S20
3-I	c.1058C>A	p.Ala353Asp	Hom	9	6.0	ND	Yes	1 affected sibling (3-II)	SRNS, cyclophosphamide resistant	Sensorineural deafness	6.5	No	NA	NA	FSGS	kidney failure	S20
3-II	c.1058C>A	p.Ala353Asp	Hom	9	2.5	ND	Yes	1 affected sibling (3-I)	SRNS	Sensorineural deafness	NA	Yes	ND	No effect on sensorineural deafness, decrease in proteinuria	FSGS	Decrease in proteinuria, severe growth retardation	S20
4	c.1058C>A	p.Ala353Asp	Hom	9	2.5	ND	Yes	ND	SRNS	Seizures, white matter abnormalities	3.4	No	NA	NA	FSGS	Died of sepsis	S20
5	c.1341G>A<br>c.1383delG	p.Trp447Ter<br>p.Gln461fs*478	Het<br>Het	11<br>12	3.0	ND	No	ND	SRNS, cyclophosphamide resistant	Sensorineural deafness	NA	No	NA	NA	FSGS	ND	S20
6	c.189_191delGAA<br>c.782C>T	p.Lys64del<br>p.Pro261Leu	Het<br>Het	2<br>7	3.8	M	ND	Healthy parents, 1 healthy sister	SRNS	Sensorineural deafness, mild weakness in lower extremities	6.0	Yes	ND	ND	cFSGS	kidney failure, kTx	S21
7	c.189_191delGAA<br>c.686A>C	p.Lys64del<br>p.Gln229Pro	Het<br>Het	2<br>6	1.9	F	ND	Healthy parents, no siblings	SRNS	Sensorineural deafness, exotropia with nystagmus on both eyes	2.6	Yes	ND	ND	FSGS	kidney failure	S21
8	c.189_191delGAA<br>c.782C>T	p.Lys64del<br>p.Pro261Leu	Het<br>Het	2<br>7	3.9	F	ND	Healthy parents, no siblings	SRNS	Sensorineural deafness	4.0	Yes	ND	ND	cFSGS	kidney failure, kTx	S21
9	c.189_191delGAA<br>c.782C>T	p.Lys64del<br>p.Pro261Leu	Het<br>Het	2<br>7	2.6	F	ND	Healthy parents, 1 unaffected sister	SRNS	Sensorineural deafness	4.5	Yes	ND	ND	cFSGS	kidney failure, kTx	S21
10	c.189_191delGAA<br>c.782C>T	p.Lys64del<br>p.Pro261Leu	Het<br>Het	2<br>7	1.25	F	ND	Healthy parents, 1 deceased sister, 1 healthy sister	SRNS	Sensorineural deafness, bilateral optic nerve atrophy	1.4	Yes	ND	ND	FSGS	kidney failure, kTx	S21

11	c.189_191delGAA<br>c.782C>T	p.Lys64del<br>p.Pro261Leu	Het<br>Het	2<br>7	2.1	M	ND	Healthy parents, 1 healthy brother	SRNS	Sensorineural deafness, mild muscle weakness in lower extremities	5.0	Yes	ND	ND	FSGS	kidney failure, kTx	S21
12	c.41G>A	p.Trp14Ter	Hom	1	16	F	ND	Mother with a history of proteinuria, FSGS. Healthy father.	Proteinuria, CKD	ND, no sensorineural deafness.	ND	Yes	ND	Decrease in proteinuria	FSGS	Decrease in proteinuria	S22
13	c.1078C>T<br>c.804delC	p.Arg360Trp<br>p.Leu269Trpfs*13	Het<br>Het	9<br>8	2	F	No	ND	SRNS	None	NA (fu 4 yrs)	Yes	30mg/kg/day	Complete remission	FSGS	Complete remission	S23
14	c.782C>T	p.Pro261Leu	Hom	7	0.7	M	No	Healthy parents	SRNS	None	1.7	Yes	ND	No neurological symptoms	MPGN type 1	kidney failure	S18
15	c.1078C>T	p.Arg360Trp	Het	9	0.8	M	ND	ND	Nephrotic proteinuria, hypoalbuminemia	Cardiovascular abnormality, motor and mental retardation, unilateral ptosis. Sensorineural deafness	NA (fu 2 yrs)	Yes	30mg/kg/day	Decrease in proteinuria, no effect on sensorineural deafness, improvement growth retardation.	ND	Complete remission	S24
16	c.1154A>C<br>c.1235A>G	p.Asp385Ala<br>p.Tyr412Cys	Het<br>Het	11<br>12	4.5	M	No	ND	SRNS	ND	ND	ND	ND	ND	MPGN	Response to CsA	S4
17	c.1058C>A	p.Ala353Asp	Hom	9	4	M	Yes	ND	SRNS	ND	ND	ND	ND	ND	FSGS	Response to CsA	S4
18	c.1058C>A	p.Ala353Asp	Hom	9	3.2	M	No	ND	SRNS	ND	ND	ND	ND	ND	FSGS	Response to CsA, cyclophosphamide	S4
19-I	c.1058C>A	p.Ala353Asp	Hom	9	7	F	Yes	Brother with sensorineural hearing loss (19-II). Father's maternal cousin with kidney failure and hearing loss. Healthy mother and other brother.	SRNS	Sensorineural deafness	8	Yes	20mg/kg/day	Treatment started after kTx. no effect on sensorineural deafness	FSGS	ESRD, kTx kidney failure	S25

19-II	c.1058C>A	p.Ala353Asp	Hom	9	10	M	Yes	Sister with sensorineural hearing loss and SRNS (19-I). Father's maternal cousin with kidney failure and hearing loss. Healthy mother and brother.	Sensorineural deafness, no kidney involvement	No neurological involvement	NA	Yes	20mg/kg/day	No effect on sensorineural deafness	NA	No kidney involvement	S25

COQ8B/*ADCK4* gene

	**Nucleotide alterations**	**Consequences at protein level**	**Hom /Het**	**Exon**	**Age at onset (y)**	**Gender**	**Consanguinity**	**Family history**	**Presenting symptoms**	**Extrarenal symptoms**	**Age at kidney failure (y)**	**CoQ_{10} supplementation**	**Dose**	**Treatment effect**	**Histopathology**	**Outcome**	**Ref**
1	c.101G>A<br>c.954_956dup	p.Trp34Ter<br>p.Thr319dup	Het<br>Het	2<br>11	10	ND	No	ND	SRNS	None	12	No	NA	NA	FSGS	kidney failure, kTx	S26
2-I	c.532C>T	p.Arg178Trp	Hom	7	9.8	M	ND*	One affected sibling (2-II)	Mild edema, hypertension	Hypermetropia, astigmatism	9.8	No	NA	NA	FSGS	kidney failure	S27
2-II	c.532C>T	p.Arg178Trp	Hom	7	14.3	M	ND*	One affected sibling (2-I)	Hypertension, polyuria, polydipsia	Hypermetropia, astigmatism	14.3	No	NA	NA	FSGS	kidney failure	S27
3-I	c.532C>T	p.Arg178Trp	Hom	7	7	ND	Yes	One affected sibling (3-II)	SRNS	ND	7	No	NA	NA	GS	kidney failure, kTx	S26
3-II	c.532C>T	p.Arg178Trp	Hom	7	13	ND	Yes	One affected sibling (3-I)	SRNS	ND	ND	No	NA	NA	FSGS	kTx	S26
4	c.532C>T<br>c.748G>C	p.Arg178Trp<br>p.Asp250His	Het<br>Het	7<br>9	0.8	F	No	Healthy parents	Proteinuria	Mental developmental retardation, delayed development of the brain	NA (fu age 1.8 yrs)	Yes	15-30 mg/kg/day	Decrease in proteinuria	Not performed	Normal kidney function	S28

5	c.625C>G<br>c.614C>T<br><br>§§§	p.Asp209His<br>p.Ser205Asn	Het<br>Het	8<br>8	11	F	No	Healthy parents	Nephrotic syndrome	None	NA (fu age 12 yrs)	Yes	15-30 mg/kg/ day	No improvement clinical symptoms	FSGS	Proteinuria, CKD	S28
6	c.625C>G<br>c.918G>T	p.Asp209His<br>p.Cys306Ter	Het<br>Het	8	14	M	ND	One sister with chronic sclerosing glomerulonephritis. Other family members healthy	Proteinuria	None	NA	Yes	150mg/ day	Partial effect, decrease in proteinuria.	FSGS	Decrease in proteinuria	S29
7	c.532C>T<br>c.748G>C	p.Arg178Trp<br>p.Asp250His	Het<br>Het	7<br>9	9.2	F	ND	Positive family history for proteinuria and/or kidney failure	SRNS	ND	11	No	NA	NA	Sclerosing glomerulonephritis	kidney failure	S30
8-I	c.645delT<br>c.1430G>A	p.Phe215Leufs*14<br>p.Arg477Gln	Het<br>Het	8<br>15	13	ND	No	One affected sibling (8-II)	SRNS	ND	13	No	NA	NA	FSGS	kidney failure, kTx	S26
8-II	c.645delT<br>c.1430G>A	p.Phe215Leufs*14<br>p.Arg477Gln	Het<br>Het	8<br>15	12	ND	No	One affected sibling (8-I)	SRNS	ND	12	No	NA	NA	FSGS	kidney failure, kTx	S26
9-I	c.857A>G<br>c.1447G>T	p.Asp286Gly<br>p.Glu483Ter	Het<br>Het	10<br>15	14	ND	No	Two affected siblings (9-II, 9-III)	SRNS	Hypertension, goiter	15	No	NA	NA	FSGS	kidney failure, kTx	S26
9-II	c.857A>G<br>c.1447G>T	p.Asp286Gly<br>p.Glu483Ter	Het<br>Het	10<br>15	3	ND	No	Two affected siblings (9-I, 9-III)	SRNS	ND	ND	No	NA	NA	FSGS	ND	S26
9-III	c.857A>G<br>c.1447G>T	p.Asp286Gly<br>p.Glu483Ter	Het<br>Het	10<br>15	9	ND	No	Two affected siblings (9-I, 9-II)	SRNS	ND	ND	No	NA	NA	FSGS	ND	S26
10-I	c.958C>T	p.Arg320Trp	Hom	11	12	ND	Yes	One affected sibling (10-II)	SRNS	Goiter	17	No	NA	NA	FSGS	kidney failure	S26
10-II	c.958C>T	p.Arg320Trp	Hom	11	20	ND	Yes	One affected sibling (10-I)	SRNS	ND	23	No	NA	NA	Not performed	kidney failure	S26

11-I	c.1027C>T	p.Arg343Trp	Hom	11	20	N D	Yes	One affected sibling (11-II)	SRNS	Dilated cardiomyopathy	20	No	NA	NA	ND	kidney failure	S26
11-II	c.1027C>T	p.Arg343Trp	Hom	11	18	N D	Yes	One affected sibling (11-I)	SRNS	ND	19	No	NA	NA	Collapsing FSGS	kidney failure	S26
12	c.1199-1200insA	p.His400Asnfs*11	Hom	13	<1	M	Yes	ND	SRNS	Neurologic developmental delay	NA	Yes	15 mg/kg/day	Disappearance of edema, decrease in proteinuria	FSGS	Decrease in proteinuria	S26
13-I	c.1356-1362delGGGCCCT	p.Gln452Hisfs*	Hom	15	16	N D	Yes	One affected sibling (13-II)	SRNS	ND	ND	No	NA	NA	Collapsing FSGS	ND	S26
13-II	c.1356-1362delGGGCCCT	p.Gln452Hisfs*	Hom	15	21	N D	Yes	One affected sibling (13-II)	SRNS	ND	ND	No	NA	NA	Collapsing FSGS	ND	S26
14-I	c.1339dupG	p.Glu447Glyfs*10	Hom	15	14	M	ND*	Four affected siblings (14-II, 14-III, 14-IV, 14-V)	Mild edema	None	17.7	No	NA	NA	FSGS	kidney failure	S27
14-II	c.1339dupG	p.Glu447Glyfs*10	Hom	15	7.3	F	ND*	Four affected siblings (14-I, 14-III, 14-IV, 14-V)	Mild edema	Epilepsy Large ASD, intermediate AVSD	12.6	No	NA	NA	FSGS	kidney failure	S27
14-III	c.1339dupG	p.Glu447Glyfs*10	Hom	15	17	F	ND*	Four affected siblings (14-I, 14-II, 14-IV, 14-V)	Mild edema	None	18	No	NA	NA	Not performed	kidney failure	S27
14-IV	c.1339dupG	p.Glu447Glyfs*10	Hom	15	27	F	ND*	Four affected siblings (14-I, 14-II, 14-III, 14-V)	Mild edema, hypertension	None	31	No	NA	NA	Not performed	kidney failure	S27
14-V	c.1339dupG	p.Glu447Glyfs*10	Hom	15	7	F	ND*	Four affected siblings (14-I, 14-II, 14-III, 14-IV)	Incidental	None	NA (fu age 12y)	No	NA	NA	Not performed	ND	S27
15-I	c.1339dupG	p.Glu447Glyfs*10	Hom	15	25.7	F	ND*	Two affected siblings	Nephrotic syndrome, CKD	None	35.4	Yes	20-30 mg/kg/day	No extra renalsymptoms, supplementation started after CKD,	FSGS	kidney failure, HD	S27, S31

								(15-II, 15-III)						no improvement kidney function.			
15-II	c.1339dupG	p.Glu447Glyfs*10	Hom	15	16.7	M	ND*	Two affected siblings (15-I, 15-III)	Nephrotic syndrome, kidney failure	Trace MI, TI	16.7	No	NA	NA	Not performed	kidney failure, kTx	S27, S31
15-III	c.1339dupG	p.Glu447Glyfs*10	Hom	15	13.5	M	ND*	Two affected siblings (15-I, 15-II)	Incidental	Mild mental retardation	16.6	No	NA	NA	FSGS/GGS	kidney failure, HD	S27 S,31
16-I	c.1339dupG	p.Glu447Glyfs*10	Hom	15	14.9	M	ND*	Three affected siblings (16-II, 16-III, 16-IV)	CKD	None	14.9	No	NA	NA	Not performed	kidney failure	S27
16-II	c.1339dupG	p.Glu447Glyfs*10	Hom	15	13.2	F	ND*	Three affected siblings (16-I, 16-III, 16-IV)	Hypertension	Epilepsy	13.2	No	NA	NA	Not performed	kidney failure	S27
16-III	c.1339dupG	p.Glu447Glyfs*10	Hom	15	18	M	ND*	Three affected siblings (16-I, 16-II, 16-IV)	CKD	None	18	No	NA	NA	Not performed	kidney failure	S27
16-IV	c.1339dupG	p.Glu447Glyfs*10	Hom	15	9	M	ND*	Three affected siblings (16-I, 16-II, 16-III)	Incidental, asymptomatic	None	NA (fu age 9.3 yrs)	Yes	30 mg/kg/day	Decrease in proteinuria	Not performed	Drop in albuminuria 80% within 6 weeks	S27
17	c.1339dupG	p.Glu447Glyfs*10	Hom	15	17.6	M	ND*	ND	CKD	None	18.0	No	NA	NA	cFSGS	kidney failure	S27
18	c.1339dupG	p.Glu447Glyfs*10	Hom	15	12	F	ND*	ND	Nephrotic syndrome	None	ND	No	NA	NA	Not reported individually	Died at 14.8 y of age	S31
19-I	c.1339dupG	p.Glu447Glyfs*10	Hom	15	12.4	F	ND*	Two affected siblings (19-II, 19-III)	Non nephrotic proteinuria, kidney failure	Hypertrophic cardiomyopathy, short stature	12.5	Yes	20-30 mg/kg/day	No improvement of kidney function	Not reported individually	kidney failure, HD	S31
19-II	c.1339dupG	p.Glu447Glyfs*10	Hom	15	9.6	F	ND*	Two affected siblings (19-I, 19-III)	Nephrotic syndrome, CKD	None	10.5	Yes	20-30 mg/kg/day	No improvement of kidney function	Not reported individually	kidney failure, PD	S31
19-III	c.1339dupG	p.Glu447Glyfs*10	Hom	15	20.3	F	ND*	Two affected siblings (19-I, 19-II)	Non nephrotic proteinuria, kidney failure	None	20.5	Yes	20-30 mg/kg/day	No improvement of kidney function	Not reported individually	kidney failure, kTx rejection, HD	S31

20-I	c.1339dupG	p.Glu447Glyfs*10	Hom	15	16.4	F	ND*	Two affected siblings (20-II, 20-III)	Nephrotic syndrome, CKD	None	17	Yes	20-30 mg/kg/day	No improvement of kidney function	Not reported individually	kidney failure, kTx	S31
20-II	c.1339dupG	p.Glu447Glyfs*10	Hom	15	6.4	M	ND*	Two affected siblings (20-I, 20-III)	Non nephrotic proteinuria, CKD	Seizure	11	Yes	20-30 mg/kg/day	No improvement of kidney function	Not reported individually	kidney failure, kTx	S31
20-III	c.1339dupG	p.Glu447Glyfs*10	Hom	15	24	M	ND*	Two affected siblings (20-I, 20-II)	During screening, non-nephrotic proteinuria	None	NA (fu age 26 yrs)	Yes	8.8 mg/kg/day	Decrease in proteinuria	Not reported individually	Decrease in proteinuria. Stable eGFR.	S31, S34
21-I	c.293T>G	p.Leu98Arg	Hom	5	5.9	F	ND*	1 affected sibling (21-II)	Incidental	Primary nocturnal enuresis	NA (fu age 14.3 yrs)	No	NA	NA	FSGS	Decrease in proteinuria	S27
21-II	c.293T>G	p.Leu98Arg	Hom	5	13.3	M	ND*	1 affected sibling (21-I)	Mild edema	Primary nocturnal enuresis	14	No	NA	NA	FSGS	kidney failure	S27
22-I	c.293T>G	p.Leu98Arg	Hom	5	13.5	F	Yes	1 affected sibling (22-II)	Moderate edema	Lupus like symptoms	16.1	Yes	20-30mg/kg/day	No improvement of kidney function	FSGS/GGS	kidney failure, kTx	S27, S31
22-II	c.293T>G	p.Leu98Arg	Hom	5	27	F	Yes	1 affected sibling (22-I)	Incidental, non-nephrotic proteinuria	None	NA (fu age 30 yrs)	Yes	9.8 mg/kg/day	Drop in proteinuria 50% within 6 weeks. Increase in proteinuria after stop CoQ_{10} treatment during pregnancy and lactation. Decrease in proteinuria after reinstitution of CoQ_{10}.	Not performed	Stable kidney function	S27, S31, S34
23	c.293T>G	p.Lys98Arg	Hom	5	9	F	Yes	ND	Non nephrotic proteinuria, microscopic hematuria	None	NA (fu age 19.3 yrs)	Yes	11.1 mg/kg/day	Decrease in proteinuria	Not reported individually	Stable kidney function	S31, S34
24-I	c.293T>G	p.Lys98Arg	Hom	5	9	M	Yes	Two affected siblings (24-II, 24-III)	Nephrotic syndrome, kidney failure	Pulmonary hypertension	9	Yes	20-30mg/kg/day	No improvement of kidney function	Not reported individually	kidney failure, HD	S31
24-II	c.293T>G	p.Lys98Arg	Hom	5	9.6	M	Yes	Two affected siblings (24-I, 24-III)	Non nephrotic proteinuria, kidney failure	Intellectual impairment	9.6	Yes	20-30mg/kg/day	No improvement of kidney function	Not reported individually	kidney failure, PD	S31

24-III	c.293T>G	p.Lys98Arg	Hom	5	32.2	M	Yes	Two affected siblings (24-I, 24-II)	During Screening, non-nephrotic proteinuria, CKD	None	NA (fu age 40 yrs)	Yes	7.3 mg/kg/day	Decrease in proteinuria. Decrease in eGFR.	Not reported individually	Decrease in eGFR.	S31, S34
25-I	c.748G>A	p.Asp250Asn	Hom	9	16.9	M	ND*	1 affected sibling (25-II)	Hypertension, headaches	None	17.4	No	NA	NA	FSGS	kidney failure	S27
25-II	c.748G>A	p.Asp250Asn	Hom	9	13.4	F	ND*	1 affected sibling (25-I)	Mild edema, hypertension, headaches	None	13.7	No	NA	NA	FSGS	kidney failure	S27
26	c.748G>C	p.Asp250His	Hom	9	10d	F	ND	Positive family history for proteinuria and/or kidney failure	Nephrotic syndrome	ND	ND	No	NA	NA	ND	ND	30
27	c.748G>C	p.Asp250His	Hom	9	1.6	F	ND	Positive family history for proteinuria and/or kidney failure	SRNS	ND	6	No	NA	NA	FSGS	kidney failure	S30
28	c.748G>T<br>c.649G>A	p.Asp250Tyr<br>p.Ala217Thr	Het<br>Het	9<br>8	5	M	No	Healthy parents. Brother with hypoplasia of the left ear pinna, an atresia of the left external ear canal with hearing loss (no proteinuria or kidney failure)	Proteinuria during screening	None	9	Yes (after kidney function was compromised)	ND	No clinical improvement	FSGS	kidney failure, KTx	S32
29	c.748G>C<br>c.1093C>G	p.Asp250His<br>p.Gln365Glu	Het<br>Het	9<br>12	6	F	ND	Positive family history for proteinuria and/or kidney failure	Proteinuria	ND	NA (fu age 12 yrs)	No	NA	NA	FSGS	Normal renal function	S30

30	c.645delT	p.Phe215Leufs*14	Hom	8	15.1	M	ND*	ND	Mild edema, hypertension	None	15.8	No	NA	NA	FSGS	kidney failure	S27
31	c.645delT	p.Phe215Leufs*14	Hom	8	14.2	M	ND*	ND	Edema, fatigue, CKD, second degree heart failure	Retinitis pigmentosa, hypospadias	15.2	No	NA	NA	FSGS	kidney failure	S27
32	c.1199-1200dupA	p.His400Asnfs*11	Hom	13	10.8	M	ND*	ND	Edema	None	15.9	No	NA	NA	FSGS – tip lesion	kidney failure	S27
33-I	c.1199dupA	p.His400Glnfs*11	Hom	13	18	M	Yes	Three affected siblings (33-II, 33-III, 33-IV)	CKD, non-nephrotic proteinuria	None	ND	No	NA	NA	Not reported individually	Deceased at 29 y of age	S31
33-II	c.1199dupA	p.His400Glnfs*11	Hom	13	12	M	Yes	Three affected siblings (33-I, 33-III, 33-IV)	CKD, nephrotic syndrome	Seizure	13	Yes	20-30 mg/kg/day	No improvement of kidney function	Not reported individually	kidney failure, HD	S31
33-III	c.1199dupA	p.His400Glnfs*11	Hom	13	2	F	Yes	Three affected siblings (33-I, 33-II, 33-IV)	During screening, nephrotic syndrome, microscopic hematuria	None	NA (fu age 5.2 yrs)	Yes	13.3 mg/kg/day	Decrease in proteinuria after initiation of CoQ$_{10}$. Increase in proteinuria after irregular use of CoQ$_{10}$.	Not reported individually	Decrease in kidney function	S31, S34
33-IV	c.1199dupA	p.His400Glnfs*11	Hom	13	7	M	Yes	Three affected siblings (33-I, 33-II, 33-III)	During screening, non-nephrotic proteinuria, microscopic hematuria	None	NA (fu age 9.2 yrs)	Yes	10 mg/kg/day	Decrease in proteinuria.	Not reported individually	Stable kidney function	S31, S34
34-I	c.1199dupA	p.His400Glnfs*11	Hom	13	13	F	ND*	One affected sibling (34-II)	Non nephrotic proteinuria, CKD	None	16	Yes	20-30 mg/kg/day	No improvement of kidney function	Not reported individually	kidney failure, kTx, graft loss after 5 years	S31
34-II	c.1199dupA	p.His400Glnfs*11	Hom	13	5	M	ND*	One affected sibling (34-I)	Nephrotic syndrome, CKD	None	13	Yes	20-30 mg/kg/day	No improvement of kidney function	Not reported individually	kidney failure, kTx	S31
35-I	c.1199dupA	p.His400Glnfs*11	Hom	13	17.7	F	Yes	Four affected siblings (35-II, 35-III, 35-IV, 35-V)	Nephrotic syndrome, kidney failure	Pericardial effusion	17.7	No	NA	NA	Not reported individually	Deceased at 21.1y of age	S31
35-II	c.1199dupA	p.His400Glnfs*11	Hom	13	4.2	M	Yes	Four affected siblings (35-I, 35-III, 35-IV, 35-V)	Non nephrotic proteinuria	None	17.5	Yes	20-30 mg/kg/day	No improvement of kidney function	Not reported individually	kidney failure, HD	S31

35-III	c.1199dupA	p.His400Glnfs*11	Hom	13	22.6	F	Yes	Four affected siblings (35-I, 35-II, 35-IV, 35-V)	Nephrotic syndrome, CKD	None	23	No	NA	NA	Not reported individually	kidney failure, HD	S31
35-IV	c.1199dupA	p.His400Glnfs*11	Hom	13	7.7	F	Yes	Four affected siblings (35-I, 35-II, 35-III, 35-V)	During screening, nephrotic syndrome	None	NA (fu age 12 yrs)	Yes	18.8 mg/kg/day	Decrease in proteinuria. After irregular use of the drug, increase proteinuria and decrease in eGFR.	Not reported individually	Decrease in kidney function	S31, S34
35-V	c.1199dupA	p.His400Glnfs*11	Hom	13	23.7	M	Yes	Four affected siblings (35-I, 35-II, 35-III, 35-IV)	During screening, non-nephrotic proteinuria	None	NA (fu age 25 yrs)	Yes	9.4 mg/kg/day	Decrease in proteinuria	Not reported individually	Stable kidney function	S31, S34
36	c.929C>T c.1493_1494CC>AA	p.Pro310Leu p.Ala498Glu	Het Het	10 15	5.1	F	ND*	ND	Incidental	Generalized seizures secondary to hypertension while on PD; PRES, inconsistent defects of visual fields, agoraphobia	13.6	No	NA	NA	FSGS - NOS	kidney failure	S27
37	c.1430G>A	p.Arg477Gln	Hom	15	17.8	F	ND*	ND	Non nephrotic proteinuria, CKD	Autism, hypothyroidism, intellectual impairment	19	Yes	20-30 mg/kg/day	No improvement of kidney function	Not reported individually	kidney failure	S31
38-I	c.449G>A c.759C>A	p.Arg150Gln p.Asn253Lys	Het Het	6 9	8.3	F	ND	One affected sibling (38-II)	Isolated proteinuria	Congenital small atrial septal defect, solid pseudopapillary neoplasm in the distal pancreas	15.0	No	NA	NA	FSGS – NOS, nephrocalcinosis grade 2	kidney failure	S33
38-II	c.449G>A c.759C>A	p.Arg150Gln p.Asn253Lys	Het Het	6 9	5.0	M	ND	One affected sibling (38-I)	Family screening, isolated proteinuria	None	10.3	No	NA	NA	Not performed, nephrocalcinosis grade 2	kidney failure	S33
39	c.737G>A c.759C>A	p.Ser246Asn p.Asn253Lys	Het Het	9 9	10.8	F	ND	ND	Isolated proteinuria	None	13.8	No	NA	NA	FSGS collapsing, nephrocalcinosis grade 2	kidney failure, kTx no recurrence FSGS	S33
40	c.737G>A	p.Ser246Asn	Hom	9	10.1	F	ND	ND	Isolated proteinuria	None	12.5	No	NA	NA	FSGS – NOS, nephrocalcinosis grade 3	kidney failure, kTx no recurrence FSGS	S33

41	c.737G>A	p.Ser246Asn	Hom	9	12.8	F	ND	ND	Isolated proteinuria, nephrocalcinosis	None	NA	Yes	30 mg/kg/day	Complete remission (to CsA)	FSGS – NOS, nephrocalcinosis grade 3	Normal kidney function	S33
42	c.737G>A	p.Ser246Asn	Hom	9	8.2	F	ND	Positive family history for proteinuria and/or kidney failure	SRNS	ND	NA (fu age 9.2 yrs)	No	NA	NA	FSGS	SRNS, normal kidney function	S30
43	c.737G>A	p.Ser246Asn	Hom	9	17.3	F	ND	Positive family history for proteinuria and/or kidney failure	Proteinuria	ND	NA (fu age 18.8 yrs)	No	NA	NA	MsPGN	Normal kidney function	S30
44	c.737G>A<br>c.1468C>T	p.Ser246Asn<br>p.Arg490Cys	Het<br>Het	9<br>15	6.9	F	ND	ND	Isolated proteinuria	Benign phylloides tumor in left breast	10.7	No	NA	NA	FSGS, nephrocalcinosis grade 2 or 3	kidney failure, kTx no recurrence FSGS	S33
45	c.241G>T<br>c.1468C>T	p.Glu81Ter<br>p.Arg490Cys	Het<br>Het	4<br>15	11	M	ND	Negative family history for proteinuria and/or kidney failure	Nephrotic syndrome	None	NA (fu age 12 yrs)	No	NA	NA	FSGS	SRNS, normal kidney function	S30
46	c.448C>T<br>c.748G>C	p.Arg150Ter<br>p.Asp250His	Het<br>Het	6<br>9	8	F	ND	Negative family history for proteinuria and/or kidney failure	SRNS	ND	11.7	No	NA	NA	FSGS	kidney failure	S30
47	c.532C>T	p.Arg178Trp	Hom	7	14	F	Yes	Two healthy brothers	CKD	Mild cognitive impairment	14.8	Yes	3dd 100 mg (= 3.7 mg/kg/day)	No recurrence proteinuria, no change in mild cognitive impairment	Not performed	kidney failure, kTx	-

Abbreviations: ASD, atrial septum defect; AVSD, atrioventricular septal defect; CKD, chronic kidney disease; CsA, cyclosporine A; d, days; F, female; FSGS, focal segmental glomerulosclerosis; fu, follow-up; HD, hemodialysis; Het, heterozygous; Hom, homozygous; kTx, kidney transplantation; M, male; m, months; MI, mitral insufficiency; MPGN, membranoproliferative glomerulonephritis; NA, not applicable, NOS; not otherwise specified; SRNS, steroid resistant nephrotic syndrome; NS, nephrotic syndrome; ND, no data; PD, peritoneal dialysis; TI, tricuspid insufficiency; y, years.

§ The patient also harbored a heteroplasmic variant in the mitochondrial genome-encoded NADH dehydrogenase subunit 1 (MT-ND1) c.3754C>A
§§ The patient also harbored a single base homozygous mutation in the *ARSB* gene c.1213+1G>A
§§§ The patient also has two heterozygous mutations in the *NPHS1* gene c.1802C>G (p.Gly601Ala), c.1339C>T (p.Glu447Lys)
ND* No individual patient data available, however in reference S27 it is stated that patients had mostly consanguineous parents and in reference S31, 80% of the patients has consanguineous parents.

Detailed description of three patients

Three patients with CoQ_{10} deficiency were identified in our tertiary referral center (Radboudumc Amalia Children's Hospital) in the Netherlands.

Patient 1 is a girl, born after an uncomplicated pregnancy of consanguineous parents, who presented at the age of 14 years. Medical history revealed a developmental language disorder with mild cognitive impairment (IQ 74), diagnosed at the age of four. Additional work-up (including cytogenetic testing and metabolic screening) for the developmental language disorder revealed no abnormalities at that time, except for a mildly elevated serum alanine level, for which no follow-up had taken place. Retrospectively, urinalysis at the age of four was performed by the general practitioner and showed proteinuria (1 g/l) for which no follow-up was undertaken either. She presented at the age of 14 years with nausea and fronto-temporal headache. On physical examination her blood pressure was elevated (145/93 mmHg) and initial laboratory evaluation showed elevated creatinine levels (262 µmol/l). Additional family history revealed CKD with unknown etiology in a distant relative. Renal ultrasound showed normal sized kidneys, increased density of the renal cortex, and diminished corticomedullary differentiation. Based on multiple urinary tract infections in the early years of life reported by the family, the differential diagnosis included CKD due to kidney dysplasia or reflux nephropathy. Nine months after presentation hemodialysis was started, and a year later the patient received a kidney transplant from a living related donor. Genetic diagnostics using whole exome sequencing with a renal disorders gene panel (286 genes) to determine the etiology of CKD showed a homozygous c.532C>T (p.(Arg178Trp) mutation in the *COQ8B/ACDK4* gene (Table S2), previously indicated to be pathogenic.[S26] She was started on CoQ_{10} supplementation (Q_{10} softgel capsules, 100 mg 3 times a day, 3.7mg/kg/day). After a few months, supplementation was discontinued at request of the patient due to complaints of heartburn, which is a known side effect of CoQ_{10}. No additional extrarenal symptoms have been identified over the years.

Patient 2 is a 6-year-old girl, born small for gestational age at 37 weeks of gestation. Pregnancy was complicated by the presence of a single umbilical artery. At two years of age, she presented at her local hospital with edema and abdominal distension. On physical examination she had a mildly elevated blood pressure (98/71 mmHg) and periorbital, pedal, and pubic edema. Laboratory investigations showed nephrotic proteinuria (6 g/l), hypoalbuminemia (0.9 g/dL), and increased lactate dehydrogenase levels (399 U/L) with normal kidney function (creatinine 0.29 mg/dL). Prednisolone, diuretics, and albumin infusions were started to treat the nephrotic syndrome and accompanying edema, but failed to improve the clinical situation. As the patient did not respond to prednisolone after 4 weeks of treatment, a renal biopsy was performed and showed collapsing FSGS and podocyte foot process effacement (Figure S1a, Figure S1b). Due to continuous worsening of her circulatory state with uncontrollable fluid retention causing respiratory insufficiency, a bilateral nephrectomy was performed 6 weeks after diagnosis and hemodialysis was started. Six months later the patient received a donor kidney from her mother. As the patient was diagnosed with SRNS, genetic analysis using whole exome sequencing with renal disorders gene panel (286 genes) was performed to detect possible disease-causing mutations. Two compound heterozygous *COQ2* mutations, c.590G>A; p.(Arg197His) and c.683A>G; p.(Asn228Ser), were found, which have previously been reported as pathogenic.[S11,S36] Subsequently, CoQ_{10} supplementation (30mg/kg/day) was initiated to prevent development of extrarenal symptoms (Table S2). Baseline mitochondrial CoQ_{10} levels in leukocytes were not available. Currently, the patient is doing well and thus far no recurrence of the nephrotic syndrome in the transplant kidney nor extrarenal symptoms have developed.

Patient 3 is a 5-year-old girl, born after an uncomplicated pregnancy. At the age of 19 months she presented with periorbital and pedal edema. Initial laboratory investigations completed the triad of a nephrotic syndrome with hypoalbuminemia (albumin 0.9 g/dL) and proteinuria (protein-to-creatinine ratio 33.8 g/10mmol), and prednisolone and diuretics were started. As she turned out to be unresponsive to prednisolone, a kidney biopsy was performed and additional immunosuppressive therapy was started. Kidney biopsy showed partial foot process effacement and signs of collapsing FSGS (Figure S1c). Eventually, the patient went into remission with cyclosporine. Lactate levels (32.4 mg/dL) were elevated at the time of diagnosis, which raised the suspicion of a mitochondrial cytopathy as a cause of the SRNS. Genetic analysis using whole exome sequencing with a renal disorders gene panel (286 genes) revealed a homozygous c.683A>G; p.(Asn228Ser) *COQ2* mutation. This mutation has previously been reported in literature as pathogenic in a patient with SRNS.[S11,S36] Initially, the patient had difficulties with the oral intake of the CoQ_{10} capsules due to the taste and size of the capsules. Therefore, she was seen and coached by a pediatric psychologist and speech therapist after which the intake of the capsules was secured. She is now doing well on CoQ_{10} supplementation (20 mg/kg/day). Four months after start of oral supplementation, CoQ_{10} levels in the leukocytes increased to normal levels after which cyclosporine therapy was discontinued. Currently, 17 months after discontinuation of the cyclosporine therapy, the patient is doing well and shows no signs of extrarenal involvement. Nevertheless, protein-to-creatinine ratios are increasing and CoQ_{10} levels in leukocytes are near the lower limit of normal. Therefore, CoQ_{10} dose is increased to 30mg/kg/day and ACE-inhibition is optimized (Figure S2).

Supplemental Table 2: Clinical characteristics of our 3 patients with a primary CoQ_{10} deficiency

	**Patient 1**	**Patient 2**	**Patient 3**
**Gender**	Female	Female	Female
**Family history**			
Consanguinity	Yes	No	Yes
Siblings	2 healthy brothers	1 healthy brother	1 healthy sister
**Kidney involvement**			
Kidney symptoms	Proteinuria (age of 4), chronic kidney failure, eGFR 25 (age of 14)	SRNS	SRNS
Age at onset	Unknown	2.5 years	19 months
Age at diagnosis	14 years	2.5 years	19 months
Kidney pathology	NA	Podocyte foot process effacement and collapsing FSGS. No significant mitochondrial abnormalities. Amorphous material below podocytes.	Partial podocyte foot process effacement and signs of collapsing FSGS. No significant mitochondrial abnormalities. Amorphous material below podocytes.
Age at start KRT<br>time after diagnosis	14 years<br>9 months after diagnosis	2.5 years<br>6 weeks after diagnosis	NA
Outcome	kidney failure, kTx LRD	kTx LRD, no recurrence of nephrotic syndrome	SRNS, in remission after treatment with cyclosporine
**Extrarenal involvement**			
Neurological evaluation	Mild cognitive impairment	Not assessed	Decreased muscle tone and poor reflexes. Otherwise, adequate, age appropriate neurological development.
Cardiac evaluation	No abnormalities	No abnormalities	No abnormalities
Ocular evaluation	Refractive error, no signs of retinitis pigmentosa	Hypermetropic astigmatism	No abnormalities
Auditory evaluation	No abnormalities	No abnormalities	No abnormalities
Muscle weakness	No<br>CK values normal	No<br>CK values normal	No<br>CK values normal
**Biochemistry data**			
Timing<br>• LDH (U/L)<br>• Lactate (mg/dL)	At diagnosis<br>• 210 (normal value <250)<br>• -	At diagnosis<br>• 399 (normal value <250)<br>• -	At diagnosis<br>• -<br>• 32.4 (normal range 7.2-18.9)
CoQ_{10} in leukocytes (nmol/UC)	Before start of supplementation:<br>• 1.05<br>(normal range 0.76 – 2.51)	After start of supplementation:<br>• 8 months: 0.78<br>• 13 months: 0.98<br>(normal range 0.76-2.51)	Before start of supplementation:<br>• 0.59<br>After supplementation:<br>• 2 months: 1.19<br>• 4 months: 1.06<br>• 7 months: 1.37<br>• 13 months: 1.23<br>• 19 months: 0.85<br>(normal range 0.76-2.51)
**Genetic analysis**	*COQ8B/ADCK4*<br>c.532C>T; p.Arg178Trp<br>(homozygous)	*COQ2*<br>c.590G>A; p.Arg197His<br>(heterozygous)<br>c.683A>G; p.Asn228Ser<br>(heterozygous)<br><br>Both parents heterozygous for 1 mutation	*COQ2*<br>c.683A>G; p.Asn228Ser<br>(homozygous)<br><br>Both parents:<br>c.683A>G; p.Asn228Ser<br>(heterozygous)

Note: conversion factors for units: Lactate in mg/dL to mmol/L, x0.111

Abbreviations: *ADCK4*, AarF Domain Containing Kinase-4; Chr, chromosome; CK, creatine kinase; CoQ_{10}, coenzyme Q_{10}; *COQ2*, coenzyme Q2; eGFR, estimated glomerular filtration rate; kTx, kidney transplantation; LDH, lactate dehydrogenase; LRD, living related donor; NA, not applicable; KRT, kidney replacement therapy; SRNS, steroid resistant nephrotic syndrome.

Fig. S1a Light microscopy image patient 2

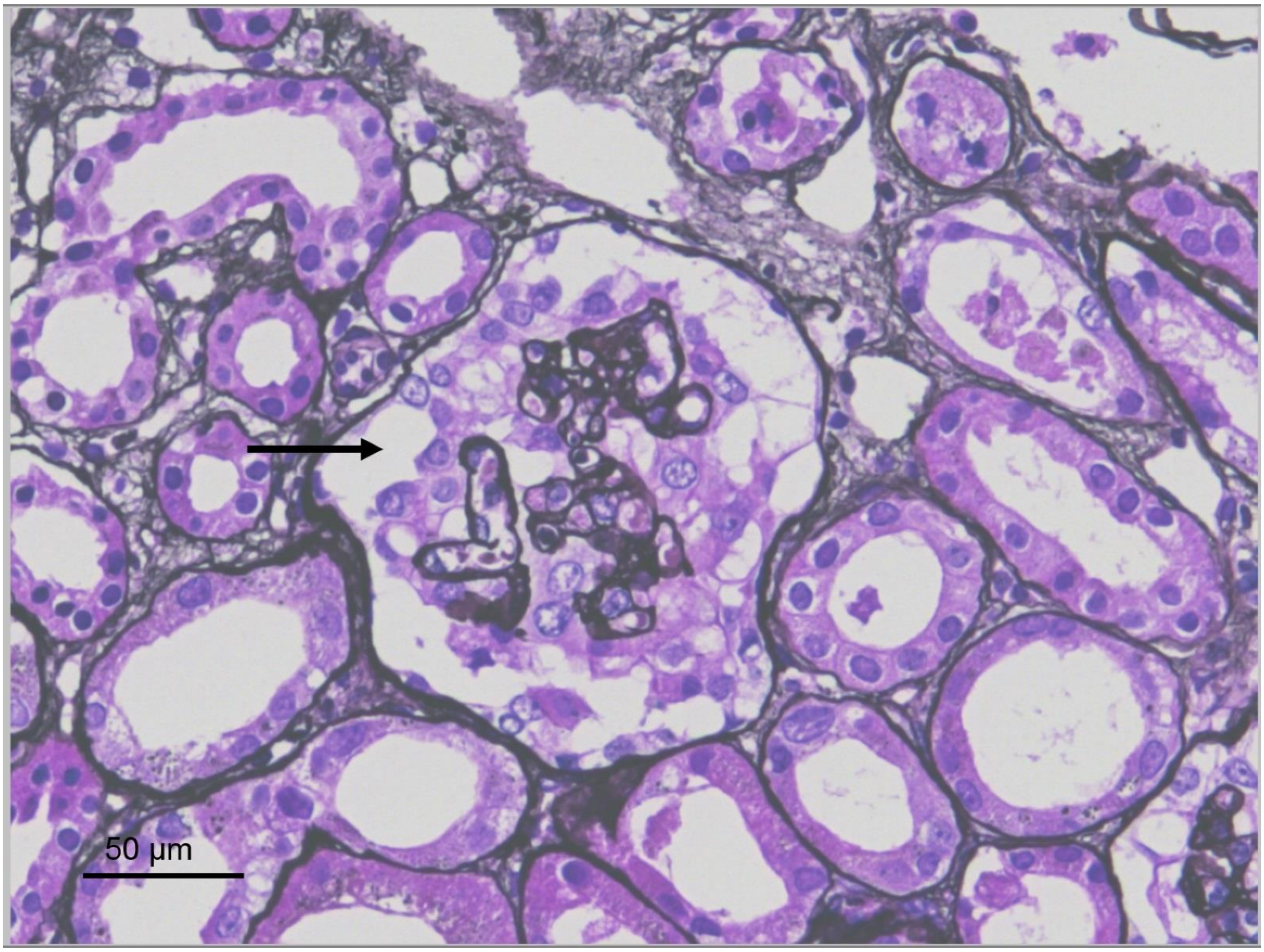

Legend:
Many glomeruli show segmental (or sometimes global) collapse of the capillaries with epithelial hyperplasia in Bowman's space, consistent with collapsing type focal and segmental glomerulosclerosis (indicated with the arrow). There are no basement membrane abnormalities. There is tubulopathy with flattening of the cells, irregular vacuolation and activated appearance of the nuclei, probably secondary to protein overload. Bar = 50µm

Fig. S1b Electron microscopy patient 2

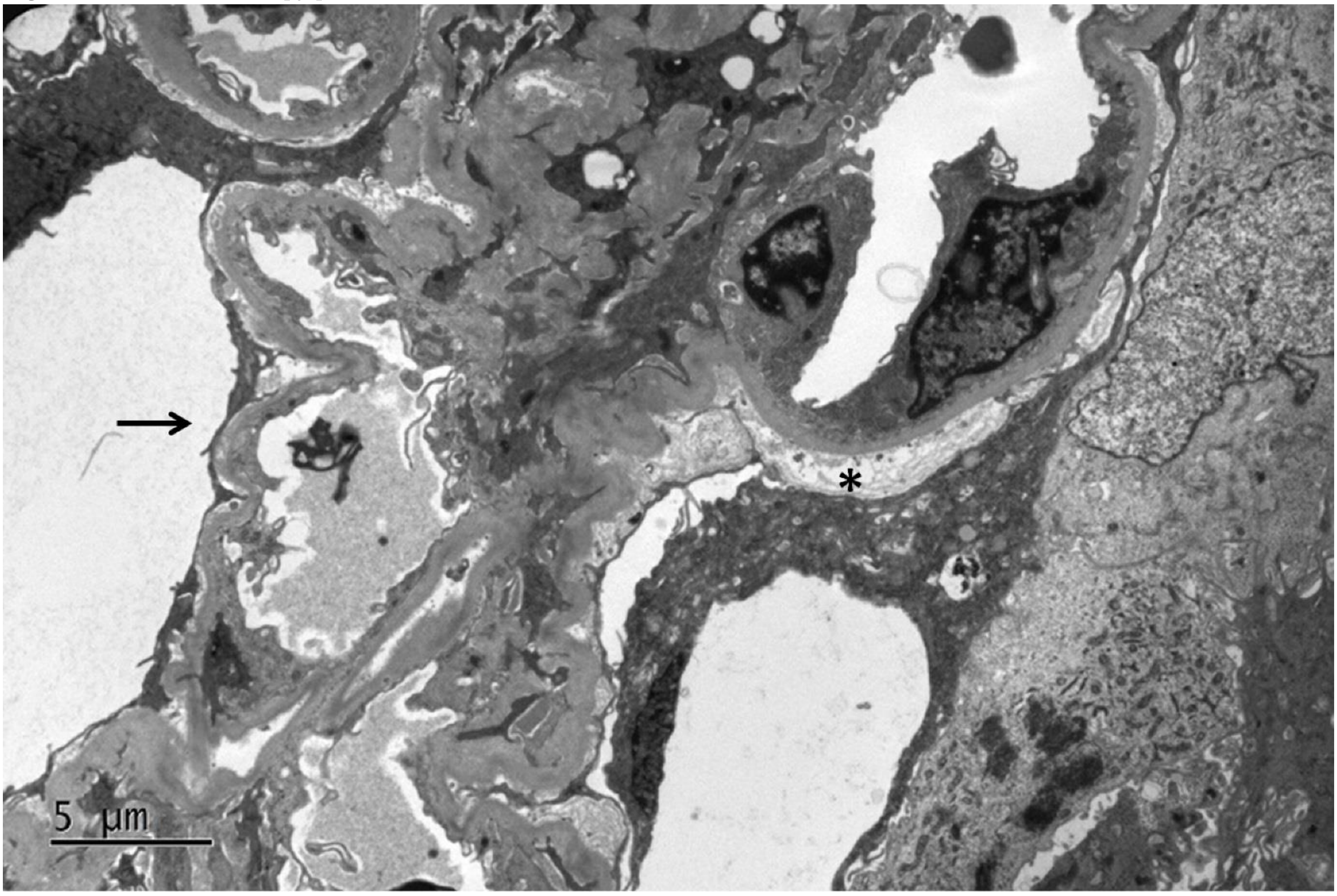

Fig. S1c Electron microscopy patient 3

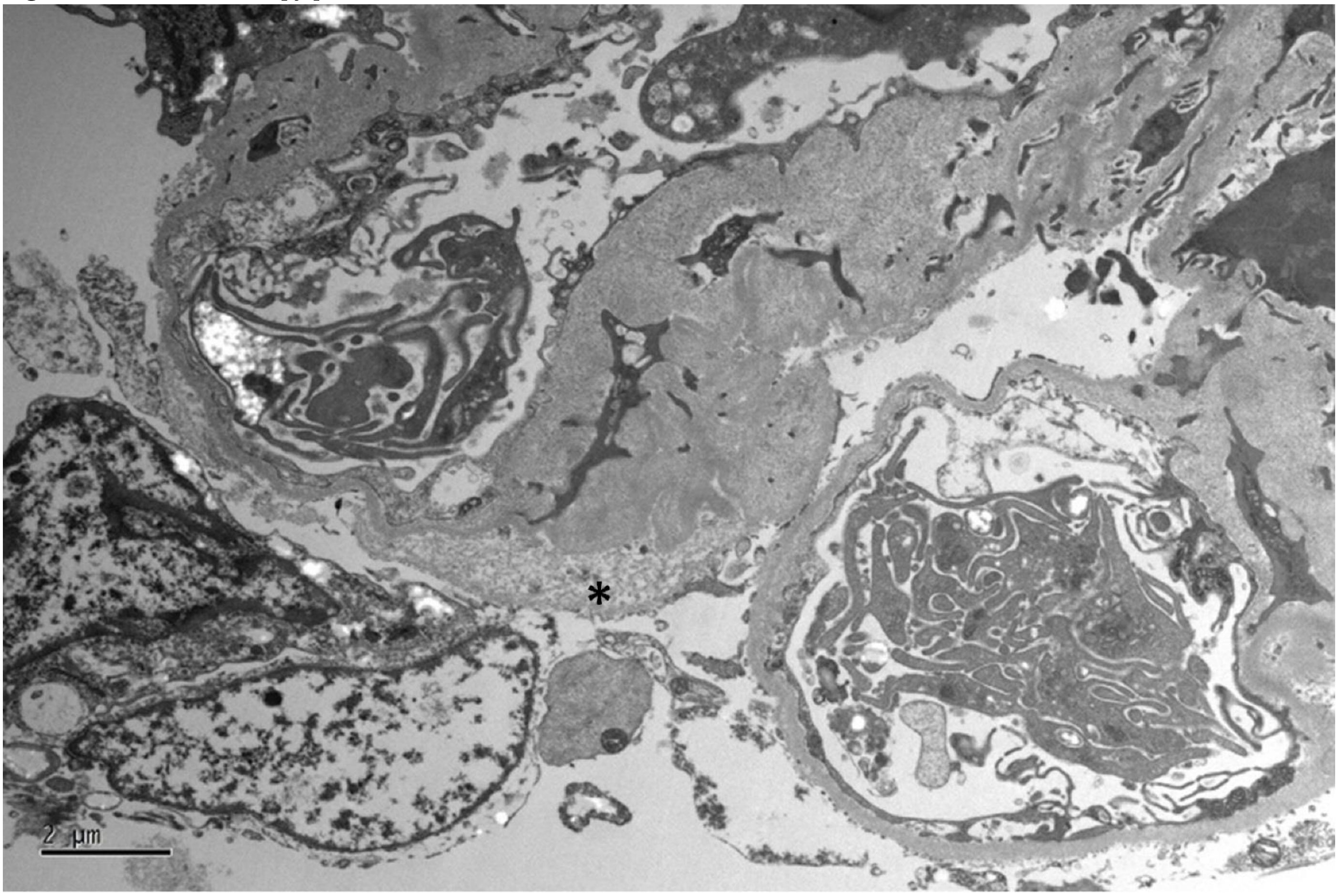

Legend:
Electron microscopy images for patients 2 and 3. There is extensive podocyte foot-process effacement (indicated with the arrow) and there are large areas of podocyte detachment from the glomerular basement membrane. Segmentally, accumulation of electron-lucent material in the subepithelial space was observed (indicated by the asterisks). This material sometimes appeared vaguely laminated but there was no evident organization. These findings were considered consistent with massive podocyte injury/collapsing focal and segmental glomerulosclerosis. Bar EM patient 2 = 5µm, bar EM patient 3 = 2µm

Fig. S2: Disease course of patient 3

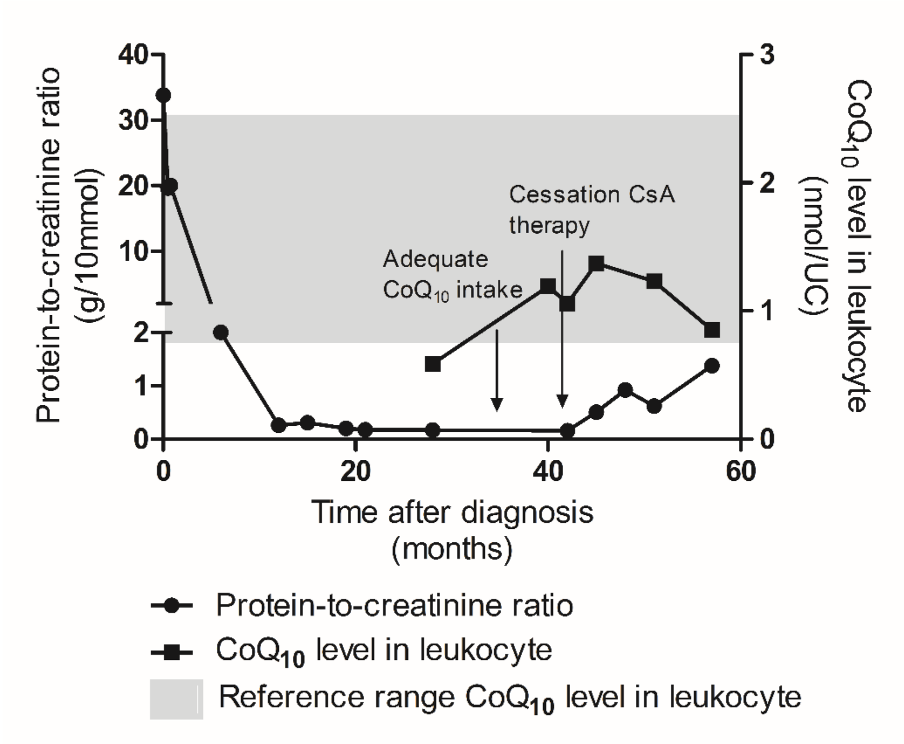

Legend:

In this graph the disease course of patient 3 is depicted. The patient went into remission with CsA treatment and after adequate CoQ_{10} intake was guaranteed, CsA was discontinued.

Abbreviations: CoQ_{10}, coenzyme Q_{10}; CsA, cyclosporine A

Supplemental references

S1. Vasta V, Merritt JL, 2nd, Saneto RP, et al. Next-generation sequencing for mitochondrial diseases: a wide diagnostic spectrum. *Pediatrics international : official journal of the Japan Pediatric Society.* 2012;54(5):585-601.DOI 10.1111/j.1442-200X.2012.03644.x

S2. Lopez LC, Schuelke M, Quinzii CM, et al. Leigh syndrome with nephropathy and CoQ10 deficiency due to decaprenyl diphosphate synthase subunit 2 (PDSS2) mutations. *Am J Hum Genet.* 2006;79(6):1125-1129.DOI 10.1086/510023

S3. Ivanyi B, Racz GZ, Gal P, et al. Diffuse mesangial sclerosis in a PDSS2 mutation-induced coenzyme Q10 deficiency. *Pediatric nephrology.* 2018;33(3):439-446.DOI 10.1007/s00467-017-3814-1

S4. Sadowski CE, Lovric S, Ashraf S, et al. A single-gene cause in 29.5% of cases of steroid-resistant nephrotic syndrome. *Journal of the American Society of Nephrology : JASN.* 2015;26(6):1279-1289.DOI 10.1681/ASN.2014050489

S5. Rotig A, Appelkvist EL, Geromel V, et al. Quinone-responsive multiple respiratory-chain dysfunction due to widespread coenzyme Q10 deficiency. *Lancet.* 2000;356(9227):391-395.DOI 10.1016/S0140-6736(00)02531-9

S6. Rahman S, Clarke CF, Hirano M. 176th ENMC International Workshop: diagnosis and treatment of coenzyme Q(1)(0) deficiency. *Neuromuscul Disord.* 2012;22(1):76-86.DOI 10.1016/j.nmd.2011.05.001

S7. Salviati L, Sacconi S, Murer L, et al. Infantile encephalomyopathy and nephropathy with CoQ10 deficiency: a CoQ10-responsive condition. *Neurology.* 2005;65(4):606-608.DOI 10.1212/01.wnl.0000172859.55579.a7

S8. Quinzii C, Naini A, Salviati L, et al. A mutation in para-hydroxybenzoate-polyprenyl transferase (COQ2) causes primary coenzyme Q10 deficiency. *Am J Hum Genet.* 2006;78(2):345-349.DOI 10.1086/500092

S9. Mollet J, Giurgea I, Schlemmer D, et al. Prenyldiphosphate synthase, subunit 1 (PDSS1) and OH-benzoate polyprenyltransferase (COQ2) mutations in ubiquinone deficiency and oxidative phosphorylation disorders. *J Clin Invest.* 2007;117(3):765-772.DOI 10.1172/JCI29089

S10. Dinwiddie DL, Smith LD, Miller NA, et al. Diagnosis of mitochondrial disorders by concomitant next-generation sequencing of the exome and mitochondrial genome. *Genomics.* 2013;102(3):148-156.DOI 10.1016/j.ygeno.2013.04.013

S11. Diomedi-Camassei F, Di Giandomenico S, Santorelli FM, et al. COQ2 nephropathy: a newly described inherited mitochondriopathy with primary renal involvement. *Journal of the American Society of Nephrology : JASN.* 2007;18(10):2773-2780.DOI 10.1681/ASN.2006080833

S12. Jakobs BS, van den Heuvel LP, Smeets RJ, et al. A novel mutation in COQ2 leading to fatal infantile multisystem disease. *J Neurol Sci.* 2013;326(1-2):24-28.DOI 10.1016/j.jns.2013.01.004

S13. McCarthy HJ, Bierzynska A, Wherlock M, et al. Simultaneous sequencing of 24 genes associated with steroid-resistant nephrotic syndrome. *Clinical journal of the American Society of Nephrology : CJASN.* 2013;8(4):637-648.DOI 10.2215/cjn.07200712

S14. Scalais E, Chafai R, Van Coster R, et al. Early myoclonic epilepsy, hypertrophic cardiomyopathy and subsequently a nephrotic syndrome in a patient with CoQ10 deficiency caused by mutations in para-hydroxybenzoate-polyprenyl transferase (COQ2). *Eur J Paediatr Neurol.* 2013;17(6):625-630.DOI 10.1016/j.ejpn.2013.05.013

S15. Desbats MA, Vetro A, Limongelli I, et al. Primary coenzyme Q10 deficiency presenting as fatal neonatal multiorgan failure. *Eur J Hum Genet.* 2015;23(9):1254-1258.DOI 10.1038/ejhg.2014.277

S16. Starr MC, Chang IJ, Finn LS, et al. COQ2 nephropathy: a treatable cause of nephrotic syndrome in children. *Pediatric nephrology.* 2018;33(7):1257-1261.DOI 10.1007/s00467-018-3937-z

S17. Xu K, Mao XY, Yao Y, et al. [Clinical analysis of one infantile nephrotic syndrome caused by COQ2 gene mutation and literature review]. *Zhonghua Er Ke Za Zhi.* 2018;56(9):662-666.DOI 10.3760/cma.j.issn.0578-1310.2018.09.006

S18. Gigante M, Diella S, Santangelo L, et al. Further phenotypic heterogeneity of CoQ10 deficiency associated with steroid resistant nephrotic syndrome and novel COQ2 and COQ6 variants. *Clin Genet.* 2017;92(2):224-226.DOI 10.1111/cge.12960

S19. Eroglu FK, Ozaltin F, Gonc N, et al. Response to early coenzyme Q10 supplementation is not sustained in CoQ10 deficiency caused by CoQ2 mutation. *Pediatr Neurol.* 2018;88:71-74.DOI 10.1016/j.pediatrneurol.2018.07.008

S20. Heeringa SF, Chernin G, Chaki M, et al. COQ6 mutations in human patients produce nephrotic syndrome with sensorineural deafness. *J Clin Invest.* 2011;121(5):2013-2024.DOI 10.1172/JCI45693

S21. Park E, Ahn YH, Kang HG, et al. COQ6 mutations in children with steroid-resistant focal segmental glomerulosclerosis and sensorineural hearing loss. *American journal of kidney diseases : the official journal of the National Kidney Foundation.* 2017;70(1):139-144.DOI 10.1053/j.ajkd.2016.10.040

S22. Song CC, Hong Q, Geng XD, et al. New mutation of coenzyme Q10 monooxygenase 6 causing podocyte injury in a focal segmental glomerulosclerosis patient. *Chin Med J (Engl).* 2018;131(22):2666-2675.DOI 10.4103/0366-6999.245158

S23. Stanczyk M, Balasz-Chmielewska I, Lipska-Zietkiewicz B, et al. CoQ10-related sustained remission of proteinuria in a child with COQ6 glomerulopathy-a case report. *Pediatric nephrology.* 2018;33(12):2383-2387.DOI 10.1007/s00467-018-4083-3

S24. Cao Q, Li GM, Xu H, et al. [Coenzyme Q(10) treatment for one child with COQ6 gene mutation induced nephrotic syndrome and literature review]. *Zhonghua Er Ke Za Zhi.* 2017;55(2):135-138.DOI 10.3760/cma.j.issn.0578-1310.2017.02.016

S25. Yuruk Yildirim Z, Toksoy G, Uyguner O, et al. Primary coenzyme Q10 Deficiency-6 (COQ10D6): Two siblings with variable expressivity of the renal phenotype. *Eur J Med Genet.* 2019.DOI 10.1016/j.ejmg.2019.01.011

S26. Ashraf S, Gee HY, Woerner S, et al. ADCK4 mutations promote steroid-resistant nephrotic syndrome through CoQ10 biosynthesis disruption. *J Clin Invest.* 2013;123(12):5179-5189.DOI 10.1172/JCI69000

S27. Korkmaz E, Lipska-Zietkiewicz BS, Boyer O, et al. ADCK4-associated glomerulopathy causes adolescence-onset FSGS. *Journal of the American Society of Nephrology : JASN.* 2016;27(1):63-68.DOI 10.1681/ASN.2014121240

S28. Feng C, Wang Q, Wang J, et al. Coenzyme Q10 supplementation therapy for 2 children with proteinuria renal disease and ADCK4 mutation: Case reports and literature review. *Medicine.* 2017;96(47):e8880.DOI 10.1097/MD.0000000000008880

S29. Yang J, Yang Y, Hu Z. A novel ADCK4 mutation in a Chinese family with ADCK4-Associated glomerulopathy. *Biochemical and biophysical research communications.* 2018;506(3):444-449.DOI 10.1016/j.bbrc.2018.10.102

S30. Wang F, Zhang Y, Mao J, et al. Spectrum of mutations in Chinese children with steroid-resistant nephrotic syndrome. *Pediatric nephrology.* 2017;32(7):1181-1192.DOI 10.1007/s00467-017-3590-y

S31. Atmaca M, Gulhan B, Korkmaz E, et al. Follow-up results of patients with ADCK4 mutations and the efficacy of CoQ10 treatment. *Pediatric nephrology.* 2017;32(8):1369-1375.DOI 10.1007/s00467-017-3634-3

S32. Lolin K, Chiodini BD, Hennaut E, et al. Early-onset of ADCK4 glomerulopathy with renal failure: a case report. *BMC Med Genet.* 2017;18(1):28.DOI 10.1186/s12881-017-0392-9

S33. Park E, Kang HG, Choi YH, et al. Focal segmental glomerulosclerosis and medullary nephrocalcinosis in children with ADCK4 mutations. *Pediatric nephrology.* 2017.DOI 10.1007/s00467-017-3657-9

S34. Atmaca M, Gulhan B, Atayar E, et al. Long-term follow-up results of patients with ADCK4 mutations who have been diagnosed in the asymptomatic period: effects of early initiation of CoQ10 supplementation. *The Turkish journal of pediatrics.* 2019;61(5):657-663.DOI 10.24953/turkjped.2019.05.003

S35. Wu X, Wang W, Liu Y, et al. A steroid-resistant nephrotic syndrome in an infant resulting from a consanguineous marriage with COQ2 and ARSB gene mutations: a case report. *BMC Med Genet*. 2019;20(1):165.DOI 10.1186/s12881-019-0898-4

S36. Salviati L, Trevisson E, Doimo M, et al. Primary Coenzyme Q10 Deficiency. 2017 Jan 26. In: Adam MP, Ardinger HH, Pagon RA, et al., editors. GeneReviews® [Internet]. Seattle (WA): University of Washington, Seattle; 1993-2018. Available from: https://www.ncbi.nlm.nih.gov/books/NBK410087/
